# Recent Advances and Future Prospects in Spark Plasma Sintered Alumina Hybrid Nanocomposites

**DOI:** 10.3390/nano9111607

**Published:** 2019-11-12

**Authors:** Nouari Saheb, Umer Hayat, Syed Fida Hassan

**Affiliations:** Department of Mechanical Engineering, King Fahd University of Petroleum and Minerals, Dhahran 31261, Saudi Arabia; umerhayat99@gmail.com (U.H.); sfhassan@kfupm.edu.sa (S.F.H.)

**Keywords:** alumina, hybrid ceramic nanocomposites, spark plasma sintering, mechanical properties, transport properties

## Abstract

Although ceramics have many advantages when compared to metals in specific applications, they could be more widely applied if their low properties (fracture toughness, strength, and electrical and thermal conductivities) are improved. Reinforcing ceramics by two nano-phases that have different morphologies and/or properties, called the hybrid microstructure design, has been implemented to develop hybrid ceramic nanocomposites with tailored nanostructures, improved mechanical properties, and enhanced functionalities. The use of the novel spark plasma sintering (SPS) process allowed for the sintering of hybrid ceramic nanocomposite materials to maintain high relative density while also preserving the small grain size of the matrix. As a result, hybrid nanocomposite materials that have better mechanical and functional properties than those of either conventional composites or nanocomposites were produced. The development of hybrid ceramic nanocomposites is in its early stage and it is expected to continue attracting the interest of the scientific community. In the present paper, the progress made in the development of alumina hybrid nanocomposites, using spark plasma sintering, and their properties are reviewed. In addition, the current challenges and potential applications are highlighted. Finally, future prospects for developing alumina hybrid nanocomposites that have better performance are set.

## 1. Introduction

**Figure 1:**
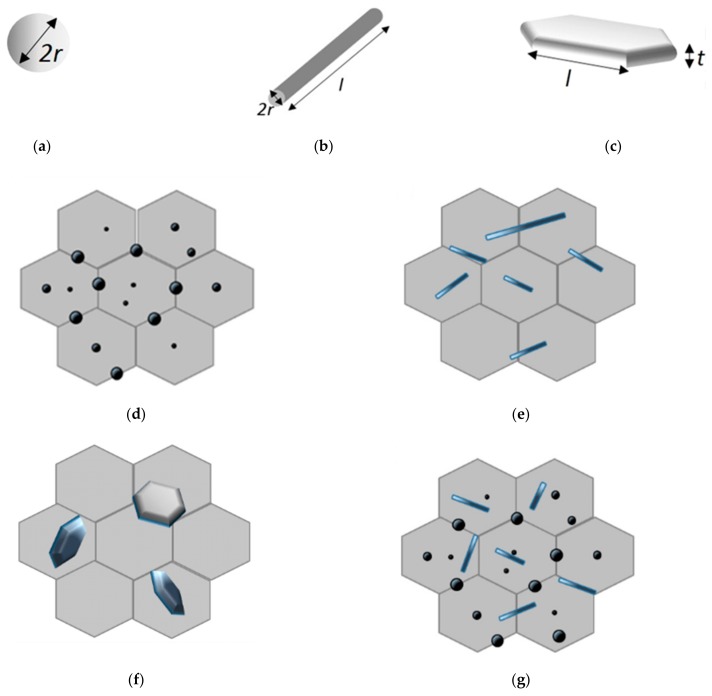
Some common nanoreinforcements and nanocomposite structures for ceramics. (**a**) zero-dimensional (0-D) round nanomaterials, (**b**) one-dimensional (1-D) needlelike-shaped nanomaterials, (**c**) two-dimensional (2-D) platelike shaped nanomaterials, (**d**) 0D nanomaterials embedded in the matrix grains, located at the grain boundaries or occupy both inter- and intra-granular positions, (**e**) 1D nanomaterials embedded in a micronic matrix, (**f**) 2D nanomaterials embedded in a micronic matrix, and (**g**) mixture of 0D and 1D nanoscale phases that are embedded in a micron-sized matrix [6].

**Figure 2:**
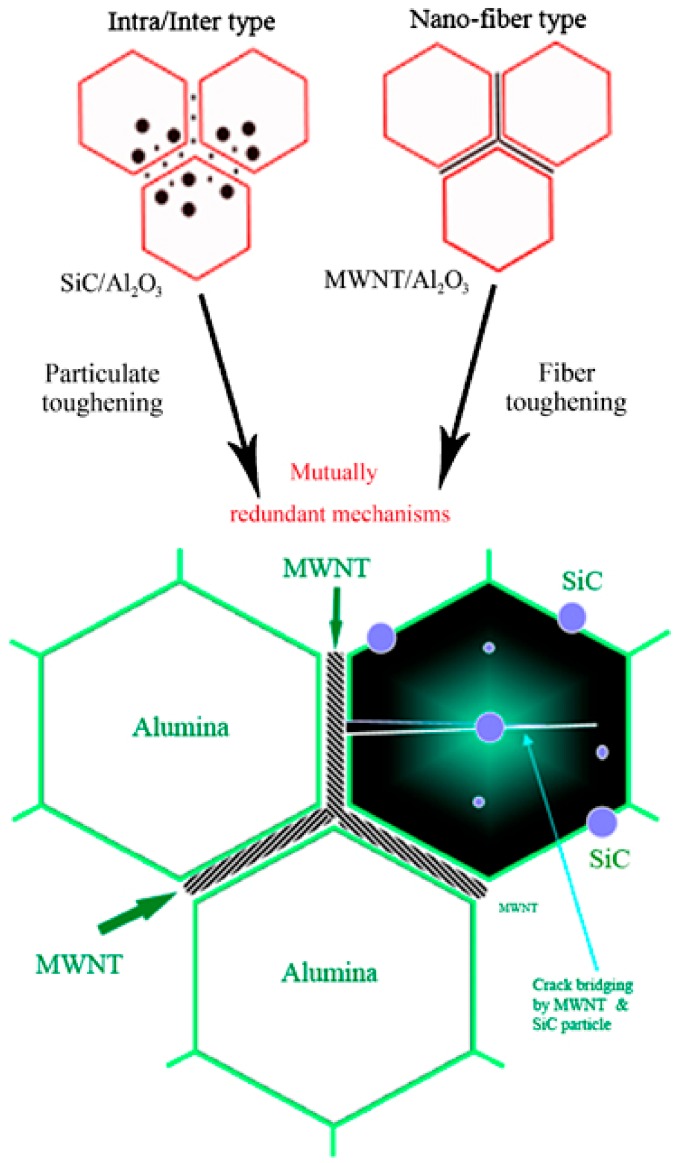
Schematic of hybrid microstructure design of alumina reinforced by carbon nanotubes (CNTs) and silicon carbide (SiC) nanoparticles (Reproduced with permission from [32]. Elsevier, 2008).

Most ceramic materials [1] have high melting temperatures, high stiffness, and high thermal stability. However, they have low fracture toughness and strength, and they show low electrical and thermal conductivities. Therefore, extensive research work has been devoted to the enhancement of the mechanical and functional properties of, ceramics through appropriate microstructure design. This includes making composites [2] and nanocomposites [3]. Ceramic nanocomposites are multiphase materials where the matrix phase is reinforced with one or more nano-phases, such that unique properties are obtained to meet or exceed design expectations [4]. As proposed by Niihara in 1991, “the concept of ceramic nanocomposites involves the adoption of the nanocomposite approach for the microstructural tailoring of structural ceramic composites” [5]. The dispersion of the nano-scale phase within the matrix grains and on the grain boundaries leads to the improvement in the mechanical, physical, and tribological properties of the nanocomposites. Figure 1 shows the most common nanoreinforcements and nanocomposite structures for ceramics. The nanoreinforcement could be: (i) zero-dimensional (0-D) round nanomaterials, such as nanoparticles and nanospheres with diameter (2*r*) less than 100 nm, (ii) one-dimensional (1-D) needlelike-shaped nanomaterials, such as fiber or tube having diameter (2*r*) less than 100 nm and aspect ratio of more than 100, or (iii) two-dimensional (2-D) platelike shaped nanomaterials, which can be layered materials with typically thickness (*t*) of the order of 1 nm and aspect ratio in the other two directions at least of 25. The 0D nanoreinforcement might be dispersed in the matrix grains, located at the grain boundaries, or occupy both inter- and intra-granular positions as shown in Figure 1d. Similarly, the 1D and 2D nanomaterials may be embedded in a micronic matrix, as presented in Figure 2e,f, respectively. Figure 2g illustrates a micron-sized matrix that is reinforced with a mixture of 0D and 1D nanoscale phases [6].

Aluminum oxide, commonly called alumina [7], is a ceramic material that is widely used in cutting tool [8] and dental applications. Moreover, it is a potential material for producing chemical and electrical insulators [9], as well as armouries [10]. However, its wide use in several structural and functional applications is limited by not only the low toughness and strength, but also the low electrical and thermal conductivities. Fortunately, reinforcing alumina with a 0D, 1D, or 2D nanoscale phase has been found to be an effective practice for developing strong and tough alumina composites with unique functionalities [11]. In this regard, alumina was reinforced by many nanoscale phases, including SiC [12,13,14,15,16,17], ZrO_2_ [18,19,20], CNT [21,22,23], and graphene [24,25,26,27,28].

Comprehensive review papers on the synthesis, processing, microstructure, properties, and performance of ceramic matrix composites, including alumina nanocomposites reinforced by SiC, ZrO_2_, CNTs, and graphene, were published [6,11,29,30,31]. Palmero et al. addressed critical issues related to the processing and properties of nanocomposite ceramics, particularly Al_2_O_3_ based composites [29]. In another review paper, Palmero [6] classified nanostructured ceramics and discussed basic structure–property relations, including the dependence of the materials properties on the grain size of the matrix and the reinforcement. Additionally, the author highlighted the influence of powder synthesis methods on the microstructure and properties of the consolidated materials. Ahmad et al. summarized advances, difficulties, and potential applications of carbon nanotube and graphene reinforced ceramic nanocomposites [11]. Galusek and Galusková [30] evaluated the influence of synthesis methods and additives on the properties of alumina-based nanocomposites. Estili and Sakka critically review the role of carbon nanotubes (CNTs) along with strengthening and toughening mechanisms, in ceramic nanocomposites [31].

Alumina hybrid nanocomposites with tailored nanostructures, improved mechanical and tribological properties, and enhanced functionalities were developed. Figure 2 presents a typical schematic of hybrid microstructure design of Al_2_O_3_ reinforced by CNTs and SiC [32]. The role of SiC particles is to strengthen alumina grain boundaries and improve the toughness. Additional fiber toughening mechanisms are due to CNTs. Moreover, CNTs and SiC have direct influence on the electrical and thermal conductivities of the hybrid composite.

Uniform distribution of the reinforcements in hybrid nanocomposite powders is a prerequisite for obtaining materials that have the anticipated properties [33]. Nanoparticles have the tendency to agglomerate and nanotubes form entangled bundles; therefore, they need to be uniformly distributed in the matrix to obtain homogenous powdered materials [30,34]. This is specifically important if a nanopowder matrix is reinforced with nanoparticles, nanotubes, and nanosheets to prepare a hybrid nanocomposite powder. Methods, such as simple wet dispersion and probe sonication [35,36], ball milling [24,32,37,38,39,40,41], molecular level mixing [21,26,42,43], sol-gel processing [44,45,46,47], and colloidal processing [22,23,35,48,49] were used to improve the dispersion of the reinforcements in alumina matrix.

Traditional sintering techniques, such as furnace sintering [36] and hot pressing sintering [35,38,50,51,52], were used to sinter alumina hybrid nanocomposites that were reinforced with SWNTs and MWCNTs [36], MWCNTs and SiCw [38], graphene nanoplatelets (GNPs) and CNTs [35], SiCW and TiC [50], Graphene and Carbon nanotube [51], and ZrO_2_ and MWCNTs [52]. Although these techniques are known for their limitation in preventing the growth of the alumina matrix, the reported values of measured properties were not far from those obtained by the spark plasma sintering method. It is believed that spark plasma sintering (SPS) will continue to be a process of choice for developing alumina hybrid nanocomposites that have preferred microstructures and novel properties [41]. This is because of the advantages of SPS over other sintering methods, which include high heating rate, low sintering time and temperature, and enhanced densification due to the role of the electric current. In addition, the process is binder-less, direct, and cost-effective. In fact, the use of SPS allowed for the sintering of alumina hybrid nancomposites to high relative density, while retaining the small grain size of the matrix. This includes Al_2_O_3_-SiC-CNTs [32,40,41,42,43], Alumina-GNPs-SiC [39], Alumina-Graphene-CNTs [53], Al_2_O_3_-SiCw-TiC [54,55], Al_2_O_3_-TiC-Ni [56], Al_2_O_3_-CNFs-SiC [57], and Al_2_O_3_-graphene based hybrid nanocomposites [58,59]. The majority of the performed studies were dedicated to the evaluation of mechanical properties and few researchers considered the tribological, electrical, and thermal properties.

As compared to composites [2] and nanocomposites [3], the development of hybrid ceramic nanocomposites is in its early stage and only limited research work is available in the literature. So far, the reported results are promising, potential applications are amazing, and the race to develop materials that have better performance is never ending. The mechanical and functional properties of ceramic nanocomposites strongly depend on: (i) the attributes of the matrix and reinforcement including, the intrinsic mechanical and physical properties, size, and dimensionality, (ii) the matrix-reinforcement interface, (iii) the degree of dispersion of the nanoscale phases, and (iv) the level of densification. In the present paper, the progress made and key issues in the synthesis and consolidation of alumina hybrid nanocomposites while using the spark plasma sintering method are reviewed. In addition, current challenges and potential applications are highlighted. Finally, future research directions for developing nanocomposites that have enhanced comprehensive performance are set.

## 2. Synthesis of Alumina Hybrid Nanocomposite Powders

### 2.1. Wet Dispersion and Sonication

**Figure 3:**
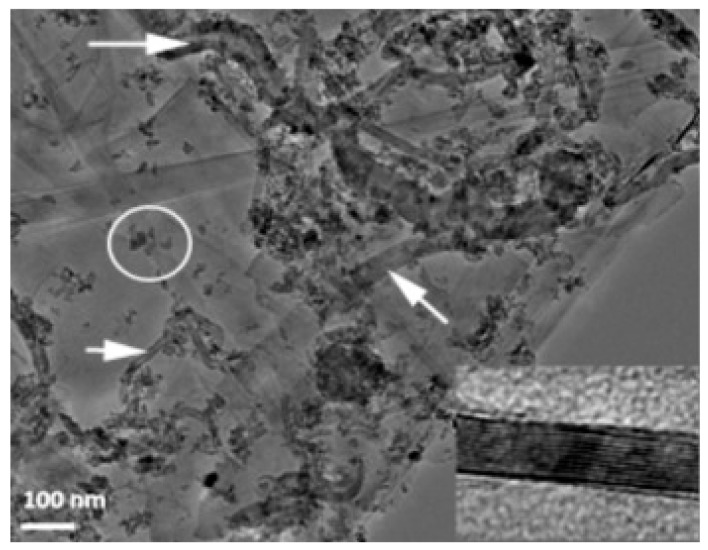
TEM image of Graphene and carbon nanotube (GNT) and Al_2_O_3_ nanopowders showing dispersed CNTs (white arrows) and Al_2_O_3_ nanoparticles (white circle) on the GNP layers. High-resolution TEM image of GNP (inset) showing stacks of 12 GNP layers (Reproduced with permission from [35]. Elsevier, 2015).

Simple wet dispersion and probe sonication were used to synthesize homogenous alumina powders that have a uniform distribution of CNTs and GNPs [35], as shown in Figure 3. It was reported that, during mixing, the CNTs that were attached to the GNP surfaces facilitated their dispersion in the alumina matrix. However, at large volume fractions, the agglomeration of the nanoreinforcements increases and the dispersion becomes more difficult.

In another investigation, Al_2_O_3_ composites containing 0.1 wt.% of MWCNTs, 0.1 wt.% SWCNTs, or 0.05 wt.% MWCNTs and 0.05 wt.% SWCNTs were prepared by sonication, drying, and grinding in an agate mortar [36]. The authors reported that the Al_2_O_3_ + 0.05 wt.% SWCNTs + 0.05 wt.% MWCNTs nanocomposite had poor hardness and fracture toughness. The low mechanical properties were attributed to the agglomeration of CNTs in the matrix, which hindered densification.

### 2.2. Ball Milling

**Figure 4:**
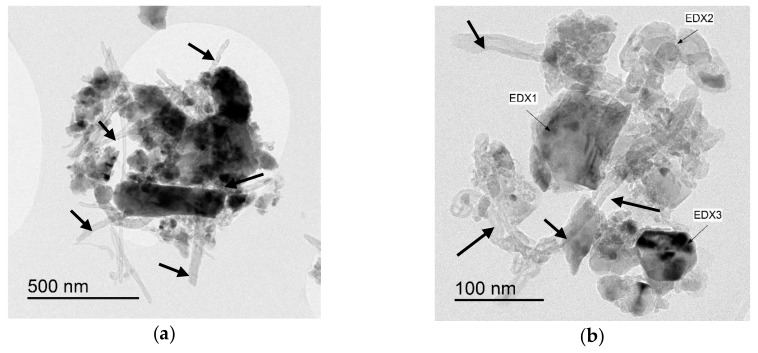
TEM images of Al_2_O_3_-5SiC-1CNT ball milled for 4 h (**a**) low and (**b**) high magnification (Reproduced with permission from [41]. Elsevier, 2016).

Ball milling [37], “a powder processing technique, which involves cold welding, fracturing, and rewelding of powder particles in a ball mill, is one of the most important techniques used to synthesize nanocomposite powders at the solid state. It can be easily used to produce composite powders at low cost with a uniform distribution of the reinforcement on the nanometre scale” [41]. The technique was used to prepare alumina composites [24] and hybrid composites [32,38,39,40,41]. A large amount of homogeneous Al_2_O_3_ composite powders containing graphene nanosheets (GNSs) were prepared, in one-step, by ball milling expanded graphite (EG) and Al_2_O_3_ powder [24]. The alumina nanoparticles acted as nanoscale milling balls to exfoliate the expanded graphite. They were also attached to the surface of delaminated graphene and helped in the dispersion of graphene. In another work, GNPs (0.38 vol.%) and SiC nanoparticles (1, 3, and 5 vol% SiC) were uniformly dispersed in alumina [39]. Other researchers used sonication and ball milling to disperse: 1 vol% of SiC nanoparticles and CNTs (0, 5, 7, and 10 vol%) [32]; 5 vol% of CNTs and SiC nanoparticles (1, 2, 3 vol%) [40] in alumina matrices. Furthermore, a relatively high fraction of SiC whiskers (25 wt.%) was dispersed in Al_2_O_3_-CNT composite powders by means of sonication and ball milling [38]. Homogenous Al_2_O_3_-SiC-CNTs hybrid nanocomposite powders were successfully synthesised while using sonication and ball milling [41]; and Figure 4 shows typical TEM images of the Al_2_O_3_-5SiC-1CNT composite powder ball milled for 4 h.

### 2.3. Molecular Level Mixing

**Figure 5:**
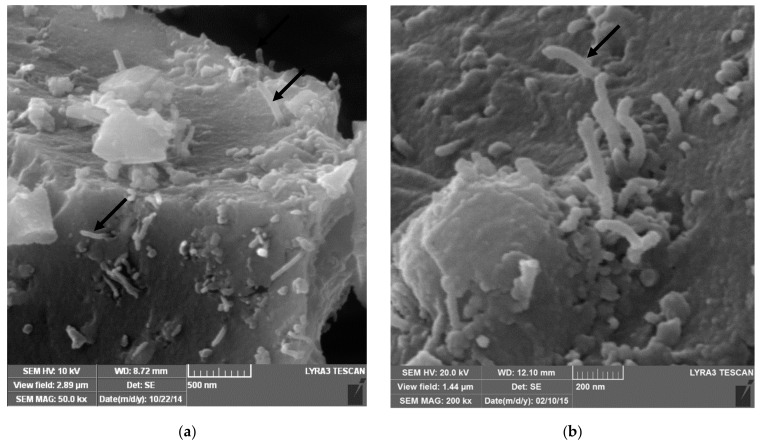
FE-SEM micrographs of Al_2_O_3_-5SiC-1CNT composite powder (**a**) low and (**b**) high magnification (Reproduced with permission from [43]. Elsevier, 2016).

The molecular-level mixing (MLM) process is a novel fabrication process that was developed to synthesize CNT reinforced metal matrix nanocomposites [42]. Its advantages include the uniform distribution of the reinforcement in the matrix and the high-interfacial strength between them due to chemical bonding. The reinforcement will be embedded inside the matrix grains rather than be present on the surface. This is of particular importance if the formation of a network structure by the reinforcement is desired. Homogenous alumina nanocomposites that were reinforced by reduced graphene oxide [26] and CNTs [21] were prepared by MLM process. The MLM was extended to uniformly disperse CNTs and SiC nanoparticles in Al_2_O_3_ matrix [43], as can be seen in Figure 5 [43]. It is believed that the MLM process could be easily used to prepare new hybrid ceramic nanocomposites, including alumina reinforced by carbonaceous materials, such as carbon nanotubes and graphene.

### 2.4. Sole-Gel Method

Sol-gel processing is an important method for synthesizing homogeneous powdered materials. In the powder case, “sol-gel refers to processing in a liquid medium to obtain a solid, which does not settle under gravity due to the formation of a diffuse network structure (the gel) comprising interparticle contacts. It allows for the synthesis of powders with a more elaborate structure from the point view of composition, purity, size, and size distribution”. The sol-gel method has been effectively used to uniformly disperse CNTs in alumina [44]. In this process, a sol containing ceramic particles is produced and the CNTs are mixed and entrapped in the gel network. This is followed by calcination as the final step. The sol-gel process was used to prepare Al_2_O_3_-CNTs nanocomposite powder [45]. “Aluminum tri-sec-butoxide was used as a precursor of alumina. Hydrolysis and peptization of aluminum hydroxide (AlOOH) led to the formation of alumina sol in which CNTs, which were dispersed in form of suspension within ethanol, were added during the gelation process” [45]. Finally, the Al_2_O_3_-CNTs nanocomposite was obtained by calcination of the dried gel powder. Recently, the sol-gel process, following a chemical route named the pyrophoric technique, was used to uniformly disperse high volume fraction of SiC particles in alumina [46]. In another investigation, aluminum oxide, titanium oxide, magnesium oxide, silicon oxide powders, and a mixture of them have been prepared by the sol-gel method for the development of advanced polycrystalline ceramics [47].

### 2.5. Colloidal Processing

The colloidal process is considered as another effective method of dispersing carbon nanotubes in ceramic matrices [22,23]. In this process, “colloidal suspensions are generally used to coat CNTs with ceramic particles by adjusting surface chemistry, stabilizing suspensions as well as lowering repulsion between CNTs which prevent agglomeration and facilitate homogeneous dispersion of CNTs throughout ceramic matrix grains” [44]. A combination of wet dispersion using a dispersant and ultra-sonication is needed to disperse the entangled CNTs. The surfaces of the ceramic particles and CNTs are usually modified by means of organic surfactants or dispersants. Practically, the effective surface charge can be altered from negative to positive while using anionic surfactants; and, the cationic surfactants can produce the opposite effect [44]. A disadvantage, of this method is the fact that the removal of surfactants or the influence of residual surfactants on the properties of Al_2_O_3_-CNTs composites were not systematically studied [22]. More recently, researchers have developed a novel surfactant less method with the aim to synthesize homogenous Al_2_O_3_-CNT composite powders containing a high volume fraction of CNTs, as high as 10%. This high CNTs loading might lead to significant improvement in electrical conductivity, as required for many functional applications. The developed surfactant-free flocculation method includes acid treatment of CNTs, flocculation, and SPS [22]. This flocculation method was effectively used to homogenously disperse up to 10 vol.% of CNTs in alumina.

**Figure 6:**
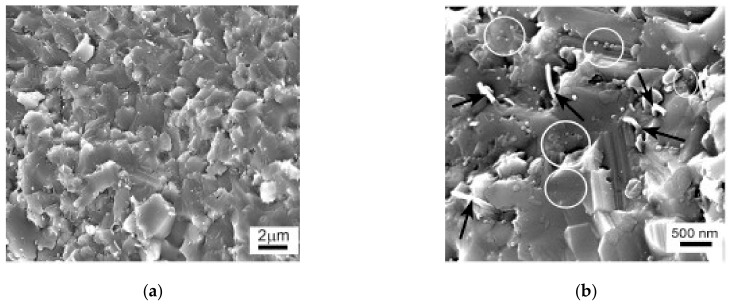
SEM images at (**a**) low and (**b**) high magnifications of fractured surfaces of the hybrid Al_2_O_3_-5SiC-2CNT nanocomposites, showing the uniform distribution of MWCNTs and SiC nanoparticles in the Al_2_O_3_ matrix. Black arrows and white circles indicate the MWCNTs and SiC nanoparticles, respectively (Reproduced with permission from [48]. Elsevier, 2016).

A water based ultrasonic assisted colloidal chemistry process [48,49] was used to prepare homogenous Al_2_O_3_-MWCNTs and Al_2_O_3_-SiC-MWCNTs hybrid nanocomposites, as can be seen in the typical SEM images presented in Figure 6 [48].

## 3. Consolidation of Alumina Hybrid Nanocomposite Powders

The fact that alumina has poor sinterability, from the one hand, and the addition of a reinforcement to produce a composite lowers the densification, from the other hand, indicates that obtaining fully dense alumina hybrid nanocomposites is challenging. Conventional consolidation techniques, such as furnace sintering [36] and hot pressing sintering [35,38,50,51,52], and non-traditional sintering techniques, such as SPS [32,39,40,41,43,53,54,55,56,57,58,59,60] and high-frequency induction heat sintering (HFIHS) [48], were used to sinter alumina hybrid nanocomposites.

Fully dense Al_2_O_3_-SiC_W_-TiC hybrid nanocomposites that have TiC particles and SiC whiskers on the grain boundaries, and TiC particles within the Al_2_O_3_ grains, were developed. The best combination of strength and toughness was obtained at 4 wt.% TiC. The maximum values of hardness, strength, and toughness were 23.9 GPa, 1200 MPa, and 7.5 MPa m^1/2^, respectively [50]. In another study [38], the Al_2_O_3_-SiC_W_-MWCNTs composites showed a sintered density of at least 99% and 60% improvement in both fracture toughness and flexural strength. In addition, CNTs were reported to reduce the wear of the highly dense Al_2_O_3_-SiC_W_-MWCNTs composites. Other researchers obtained almost fully dense (98%) Al_2_O_3_ reinforced by GNPs and CNTs. The monolithic alumina and hybrid nanocomposites had fracture toughness values of around 3.5 and 5.7 MPa m^1/2^, respectively. The flexural strength increased from 360 MPa (monolithic alumina) to 424 MPa (Al_2_O_3_-0.5wt% GNPs-1 wt% CNTs) [35]. In other study, it was reported that reinforcing Al_2_O_3_ by 0.3wt.% GNPs and 1 wt.% CNTs led to an 86% reduction in the wear rate [51]. This was attributed to the formation of a tribofilm on the worn surface due to GNPs exfoliation. The fracture toughness also increased because of the presence of CNTs. The incorporation of 1 vol% of CNTs into monolithic Al_2_O_3_ and Al_2_O_3_-ZrO_2_ composite [52] was found to increase the fracture toughness by 8% and 35%, respectively, in comparison to the monolithic Al_2_O_3_. The electrical conductivity increased from 10^−12^ S/m (Al_2_O_3_) to 2.7×10^−1^ S/m for the composites containing 2 vol% of CNTs [52]. In another work, Al_2_O_3_ hybrid nanocomposites that were reinforced by MWCNTs and SWCNTs showed lower hardness and fracture toughness when compared to Al_2_O_3_ because of the inhomogeneous dispersion of CNTs in the matrix [35]. High-frequency induction heat sintering developed dense Al_2_O_3_-MWCNTs-SiC hybrid nanocomposites with fine microstructure and substantial increase in fracture toughness (110%) and hardness (30%) when compared to pure alumina [48]. The superior hardness was attributed to the fine microstructure and the hard SiC nanoparticles, while the improvement in toughness was believed to be due to the toughening mechanisms that were imparted by the two reinforcements [48].

## 4. Spark Plasma Sintering Method

The use of the novel SPS method allowed for the sintering of composite materials to high relative density while preserving the small grain size of the matrix [41]. Therefore, SPS, as is binder-less, direct, and cost-effective process will continue to be a method of choice to develop alumina hybrid nanocomposites that have preferred microstructures and novel properties. This is due to the advantages of SPS, over other sintering methods, which include enhanced densification, high heating rate, short sintering time, and low sintering temperature.

### 4.1. The SPS Process

**Figure 7:**
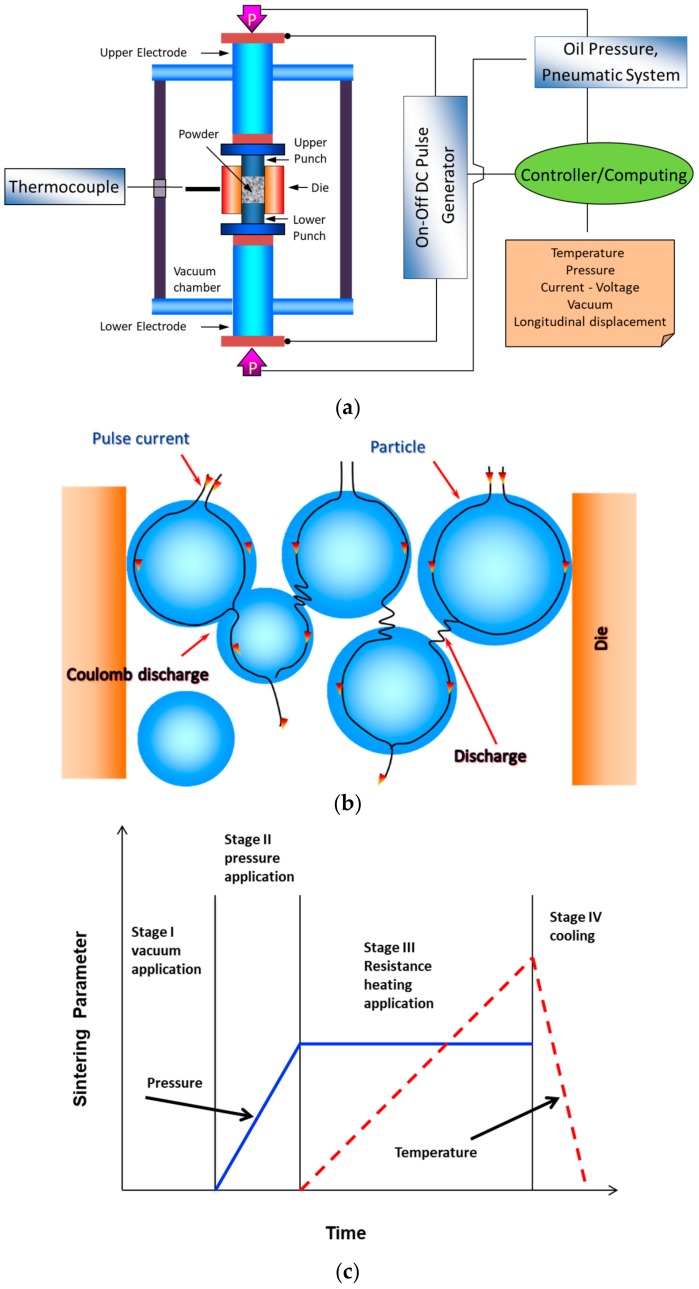
The spark plasma sintering (SPS) process (**a**), DC pulse current flow through the particles (**b**), and sintering stages (**c**) [61].

The SPS is a sintering method where a pressure and a direct electrical current are concurrently applied to consolidate powdered materials, as shown in Figure 7a [61]. This single step, binder-less, and cost-effective process has been heavily used to produce highly dense materials, with controlled microstructures and improved properties. Its advantages include, but are not limited to: (i) high heating rate, (ii) enhanced diffusion and densification, and (iii) very limited grain growth because of the reduced sintering time and temperature [61,62,63,64]. It is believed that when a spark discharge appears in a gap or at the contact point between the particles of a material, as illustrated in Figure 7b, a local high temperature-state is momentarily generated. This causes evaporation and melting on the surface of powder particles and necks are formed around the area of contact between particles. The application of pressure and current, as shown in Figure 7c, in addition to the high localized temperatures generated through resistance pulse heating, improve the heating rates and reduce sintering time and temperature and lead to the consolidation of nanopowders without excessive grain growth [61].

The SPS has attracted the interest of the scientific community and many review papers, highlighting the basic concepts, importance, and feasibility of the technique were published. The important features of the SPS process and their roles and influence on densification and properties of materials have been critically examined and reviewed by researchers [61,63,65]. Despite the fact that the SPS was deeply investigated [66,67,68,69,70] and heavily used to sinter different materials, including alumina-based composites [71,72,73], the underlying sintering mechanisms are still not fully understood. This was attributed to the complex mechanical, electrical, and thermal phenomena that are associated with SPS.

The occurrence of discharge and the existence of spark plasma in SPS remained highly debatable. Zhang and co-workers [62] proved the presence of spark discharging and the existence of plasma in SPS. However, Dustin Hulbert and co-workers [66], who investigated the existence of plasma during sintering, proved the absence of arcing, sparking, and plasma in the whole cycle of sintering. Kieback [67] believed that SPS is similar to hot pressing (HP) and the only deference is the high heating rates that were attained through the application of the current. Moreover, he “questioned the importance of unconfirmed electrical effects such as sparks, plasma, heat diffusion, electromigration, or electron wind because of lack of experimental evidence” [67]. Other researchers [68] proved that the Branly effect might occur at the beginning of sintering and results in the formation of melting zones between particles, which enhances densification. Hitchcock and co-workers investigated the role of sparks and plasma [74], and they examined the SPS process and confirmed the absence of sparking and plasma. Despite controversies among researchers regarding the SPS process, all of them appreciated the efficiency of the process to consolidate powdered materials, including hybrid nanocomposite powders, into dense materials that have preserved microstructures and novel properties.

### 4.2. Densification

The relative density is frequently used to quantity the level of densification. Moreover, it is one of the indirect indicators for assessing the dispersion of reinforcements in ceramic matrix nanocomposites. In general, high densification and good dispersion lead to better mechanical and functional properties [35]. The use of SPS allowed for the sintering of alumina hybrid nanocomposites to either near-theoretical density or high relative density values despite the poor sinterability of alumina and the fact that the presence of the reinforcement in a ceramic matrix reduces the densification [41]. For materials sintered by SPS, the density depends on temperature according to the following equation [75]:

(1) $$\left(\rho =s(\frac{T}{T_{m}})+b\right)$$

where *ρ* is the relative density, *s* is the temperature sensitivity, *T* is the sintering temperature, and *T_m_* is the melting temperature [75].

During sintering, the applied pressure helps in breaking down the agglomerates and the rearrangement of particles, which increases the sintering driving force. In sintering methods involving the use of external pressure, such as SPS, the sintering driving force depends on pressure, as follows [75]:

(2) $$\frac{d\rho }{(1-\rho )dt}=B(g\frac{\gamma }{x}+P)$$

where *ρ* is the relative density, *B* is a term that includes the diffusion coefficient and temperature, *g* is a geometric constant, *γ* is the surface energy, *x* is a parameter that represents a size scale (and hence is related to particle size), *t* is time, and *P* is the applied external pressure [75].

On the other hand, nanopowders are known for their high tendency to sinter because of not only the effect of curvature [4], but also the high concentration of equilibrium vacancies in a nanoparticle that might be expressed by the following equation:

(3) $$X_{V}^{Total}=\exp(-\frac{\Delta G_{v}^{bulk}}{k_{B}T})\exp(-\frac{\Omega \gamma }{rk_{B}T})$$

where $\Delta G_{v}^{bulk}$ is the equilibrium Gibbs free energy change for the formation of vacancies in the bulk, Ω is the atomic volume, *γ* the surface energy, *r* the radius of curvature, *K_B_* is the Boltzmann constant, and *T* is temperature [4].

The Joule heating which results from the applied current can be quantified, as follows [76]:

(4) $$I_{RMS}=\sqrt{\frac{1}{\tau }\int_{t}^{t+\tau }I^{2}t(dt)}$$

where *τ* is the sampling time and *I* is the current.

## 5. Mechanical Properties

The purpose for reinforcing alumina with hybrid nanoreinforcements has been mainly to improve its mechanical and physical properties. The enhancement in mechanical properties is credited to the outstanding mechanical characteristics of the reinforcements, small grain size of the alumina matrix, the change in the fracture mode, and the toughening mechanism that is associated with the reinforcements. Furthermore, the excellent physical properties of the reinforcements contribute to the improvement of the electrical and thermal properties of alumina. For example, SiC is known to have a hardness of approximately 30 GPa as compared to the hardness of alumina, which is approximately 17.65 GPa [46]; CNTs have high stiffness of around 1 TPa [76,77] and tensile strength up to 60 GPa [78]. Furthermore, graphene, a two-dimensional material consisting of sp^2^-hybridized carbon atoms, which is believed to be the strongest material, has exceptional mechanical properties. A perfect single-layer graphene has a stiffness of 1.0 TPa and fracture strength of 130 GPa [79]. In contrast to monolayer graphene, graphene nanosheets or graphene nanoplatelets have been found to possess outstanding mechanical properties [80,81,82]. The stiffness of GNPs with a thickness of 2–8 nm is reported to be around 0.5 TPa [83]. The fracture toughness of graphene was found to be equal to 4 MPa m^½^ [84]. As for thermal properties, at 300 K, CNTs have electrical conductivity of around 10^6^ S/m for SWNT and >10^5^ S/m for MWNT [85,86]. Moreover, they have very high thermal conductivity [87,88], with room temperature measured values of 3000 and 3500 W/mK for MWCNTs [89] and SWCNTs [90], respectively. Nevertheless, a value of 5300 W/mK was reported for the room temperature thermal conductivity of single layer graphene [91].

### 5.1. Hardness and Strength

The grain size strongly influences the properties of polycrystalline materials; therefore, the inhibition of grain growth, which might occur during sintering (as indicated in Equations (5) and (6)) [69], and design of materials with fine microstructures remain important ways to develop materials with improved mechanical properties.

(5) $$d^{n}-d_{0}^{n}=Kt$$

(6) $$K=K_{0}\exp(\frac{-Q}{RT})$$

where *d*_0_ and *d* are the grain sizes at an initial time *t*_0_ and a dwell time *t*, respectively. *K* is a temperature dependent material constant that is usually expressed with the following Arrhenius equation, *Q* is the activation energy for grain growth, *R* is the gas constant, and *T* is temperature [69].

In this regard, the reinforcements inhibit the matrix grain growths according to the Zener-type models [6].

(7) $$d=\frac{4r}{3f}$$

where *f* and *r* are the volume fraction and radius of the reinforcement, respectively, and *d* is the grain size of the matrix.

The use of hybrid nanoreinforcements and spark plasma sintering method has enabled researchers to develop alumina hybrid nanocomposites that have a small grain size of the alumina matrix. Proper selection of SPS process parameters, such as sintering pressure, temperature, and time, and heating rate minimize grain growth. Additionally, the presence of reinforcements restricts the growth of alumina grains due to the pining effect. The yield strength (*σ_y_*) and Vickers microhardness (*H_v_*) of nanomaterials depend on the average grain size according to the well-established relationships and they can be expressed, as follows [6]:

(8) $$\sigma_{y}=\;\sigma_{0}+\;kd^{-1/2}$$

(9) $$H_{v}=H_{0}+kd^{-1/2}$$

where *H*_0_ is the intrinsic hardness [92], *τ*_0_ is a material constant [93], *k* is a strengthening coefficient, and *d* is the average grain size.

The hardness [94] and flexural [95] tests are usually used to determine Vickers hardness and flexural strength, respectively, according to the following equations:

(10) $$H_{V}=1.854\frac{P}{d^{2}}$$

where *P* and *d* are the load and diagonal length, respectively.

(11) $$\sigma_{f}=\frac{3QL}{2lh^{2}}$$

where *Q* is the failure load, *L* is the span, *l* is the width, and *h* is the height.

Equations (8) and (9) suggest that improvements in hardness and strength require refinement of the microstructure and the inhibition of grain growth. Furthermore, the refinement of the microstructure contributes to the decrease in the material flaw size formed during processing, which leads to increased strength. This can be understood through the Griffith equation, which outlines the mechanical behaviour of ceramics and relates the fracture toughness and strength, as follows:

(12) $$\sigma_{f}=\frac{K_{1C}}{Y\sqrt{\pi a}}$$

where *σ_f_* is the strength, *K_IC_* is the fracture toughness, *a* is the flaw size, and *Y* is a geometric factor that is approximately equal to 1 [96].

Griffith equation reveals that a decrease in flaw size and an increase in fracture toughness are required in order to increase strength. In spark plasma sintered alumina hybrid nanocomposites, processing using SPS leads to refined microstructures, which contribute to the decrease in the material flaw size. Furthermore, the addition of hybrid nanoreinforcements increases the fracture toughness because of the induced toughening mechanisms.

### 5.2. Fracture Toughness

In addition to increasing the strength and hardness, reinforcing ceramic materials by nanoparticles, nanotubes, and nanoplatelets was found to improve the fracture toughness. For brittle materials, fracture toughness (*K_IC_*) [97] is related to stress and crack length according to the Griffith’s formula obtained by rearrangement of Equation (12), as follows:

(13) $$K_{1C}=\sigma_{f}Y\sqrt{\pi a}$$

where *σ_f_* is the stress, *a* is the half crack length, and *Y* is a constant.

The fracture toughness of alumina-based nanocomposites was evaluated while using different methods. This includes Vickers indentation fracture (VIF) [41,43,48,49,55,56,57,98,99], indentation strength in bending (ISB) [49], and single edge notched beam (SENB) [32,40,49,53,99,100] methods.

In the VIF method, the Anstis’ equation is used to evaluate the fracture toughness [101]:

(14) $$K_{1c}=0.016\sqrt[{}]{\frac{E}{H}}\frac{P}{C^{\frac{3}{2}}}$$

where *E* is the elastic modulus, *H* is the Vickers hardness, *P* is the applied load, and *C* is the diagonal crack length [101]. The elastic modulus of the composite is calculated while using the rule of mixture.

In the ISB method, a diamond Vickers indenter of a hardness tester is used to make an indent at the middle of the sample. Subsequently, the three point bending test is performed on the indented sample and the fracture toughness is calculated while using the following equation [102]:

(15) K1c=0.59(EH)18(σP13)3/4

where *E* is the Young’s modulus, *H* is the hardness, *σ* the flexural strength in the presence of the Vickers indent, and *P* the load at failure [102].

In the SENB method, the three-point bending test is performed and the fracture toughness is calculated, as follows [103]:

(16) K1C=3PmaxL2BW32α12γ

where *P* is the breaking load during three-point bending test, *L* is the bending span, *B* is the specimen breath, *W* the specimen height and *a* is the notch depth, *α* is the ratio between *a* and *W*, and *Y* is the calibration factor calculated by the equation that is given below [103]:

(17) Y=1.99−α(1−α)(2.15−3.93α+2.7α2)(1+2α)(1−α)32

Being simple and easy to use, the VIF method has been widely applied to calculate the fracture toughness; however, it was strongly criticized because it is unreliable, inaccurate, and imprecise. In addition, it is not a standard test method that is recognized by international professional societies [44,104]. Therefore, the single-edged-notched-beam (SENB) method is more accurate and reliable when compared to VIF to measure the fracture toughness.

### 5.3. Wear and Friction

The friction and wear behaviors of ceramic matrix nanocomposites are critical parameters for tribological applications. Indeed, the most important factors for understanding the tribological behavior of materials are the coefficient of friction (μ) and specific wear rate. The latter is usually calculated while using the following equation [105].

(18) $$W=\frac{V}{LF}$$

where *V*, *F*, and *L* are the wear volume, applied load, and sliding distance, respectively.

## 6. Physical Properties

The discovery of CNTs and GNPs has encouraged researchers to develop conductive polymer matrix composites (PMCs) [106]. The successful production of PMCs and their use in many practical applications has stimulated scientists to incorporate C-nanofillers in metallic matrices for improving their mechanical, physical, thermal, electrical, and magnetic properties [107]. However, extensive progress has not been thoroughly made to develop conductive ceramic composites (CCMs) and hybrid composites.

### 6.1. Electrical Conductivity

The electrical conductivity of a material can be obtained while using the dc-four probe method, and quantified according to the following equation:

(19) $$\sigma =\frac{L}{RA}$$

where *σ* is the conductivity, *L* the distance between the internal electrodes, *A* is the cross-section area of the sample, and *R* is the resistance.

For composite materials and, according to the classical percolation theory, the electrical conductivity as function of the conductive reinforcement content can be expressed by a scaling law [106,108,109,110], as follows.

(20) $$\sigma \;=\;\sigma_{0}t(p-p_{c})$$

where *P*_c_ is the percolation threshold of the conductive composite, *P* is the reinforcement content, *σ*_0_ is a scaling factor and *σ* is the conductivity [108], and *t* is an exponent that is related to the dimensionality of the conductive network within the composite.

### 6.2. Thermal Conductivity

In a single step and from a single measurement thermal conductivity, thermal diffusivity and specific heat capacity can be determined based on the theory of the Transient Plane Source technique [111] according to the ISO standard (ISO/DIS 22007-2.2). In addition, the flash method could be used to measure thermal diffusivity according to ASTM standard E1461, and the thermal conductivity is then calculated from the measured thermal diffusivity, specific heat, and bulk density, while using the following standard equation.

(21) $$K=\kappa \rho C_{p}$$

were (*κ*) is the thermal diffusivity, *C_p_* is the specific heat, and *ρ* is the density of the material.

## 7. Spark Plasma Sintered Alumina

Gurt Santanach and co-workers investigated the influence of various sintering process parameters i.e., dwell temperature, applied pressure, dwell time, and pulse pattern on spark plasma sintered monolithic Al_2_O_3_ [112]. The authors used alumina powder with an average particle size of 0.14 µm and heating rate of 100 °C/min. Investigation of density and grain size of spark plasma sintered alumina at various sintering parameters showed two regimes: densification without grain growth that occurred at low temperatures and grain growth without further densification that occurred at higher temperatures with threshold from 1100 °C to 1200 °C. Increasing the dwell temperature from 600 °C to 1100 °C using same pressure and time (100 MPa, 5 min.) increased the density from 54.6% to 94.5% without significant grain growth, but further increase in temperature from 1100 °C to 1500 °C, although, increased density from 94.5% to 99.2% it increased grain size from 0.2 to 7.6 µm. Increasing the pressure from 10 to 100 MPa while keeping temperature and time constant (1500 °C, 3 min.) favored the grain growth from 4.3 to 7.6 µm while the density almost remained same at 99.2%. This grain growth was attributed to the fact that high temperature and pressure favored grain growth through grain boundary diffusion. Increasing the dwell time from 0 to 60 min. while keeping temperature and pressure constant (1100 °C, 100 MPa) increased the density from 90.8% to 99.8% with small grain growth. Thus, dwell time affects the proportion of porosity in specimens and can be used to control it.

The effects of grain growth inhibitor and different sintering parameters i.e., temperature, holding time, heating rate, pressure on the densification, grain growth, hardness, and fracture toughness of spark plasma sintered alumina was also reported [113]. Alumina powder, with an average particle size of 0.4 µm, was used and 0.1% MgO was used as a grain growth inhibitor in few samples. It was reported that increasing the temperature from 1250 °C while leaving heating rate, sintering time, and pressure unchanged at values of 150–200 °C/min., 5 min., 50 MPa, respectively, resulted in fully dense sample either with or without MgO, but the grain growth exponentially increased and it was more prominent in samples without MgO. The hardness started to decrease from 21 GPa at 1250 °C to 16 GPa at 1500 °C while the fracture toughness almost remained constant. Increasing time from 0 to 40 min. while leaving other parameters constant i.e., 120 °C/min., 1200 °C, 50 MPa led to the increase of density from 95.8% to 100% at 20 min. and above while grain growth increased at higher holding times reaching a maximum value of 2 µm at 40 min. Hardness and fracture toughness remained almost the same within the range of 20–21 GPa and 3.2 ± 0.5 MPa m^1/2^. An increasing pressure resulted in higher grain growth in samples that were sintered at temperature greater than 1200 °C than those sintered at temperature lower than 1200 °C and fully dense samples were obtained at all pressures greater than 50 MPa when temperatures 1200 °C and above were used with other parameters i.e., 150–200 °C/min., 3 min. remaining unchanged. Increasing heating rate from 50 to 600 °C/min. while using two different temperatures 1300 and 1400 °C without holding time at 50 MPa resulted in dense compacts with relative density greater than 99.5%. Grain growth was found to be more prominent in samples that were sintered at 1400 °C than 1300 °C. Grain growth decreased, while harness increased and fracture toughness remained same as heating rate increased.

Wang and co-workers [114] studied the effect of particle sizes of starting powder, sintering parameters, and thickness of sintered samples on microstructure and densification of alumina consolidated by SPS. The authors used alumina powders with four different particle sizes (0.33, 0.4, 3.46, and 21.4 µm). When these powders were sintered at a constant heating rate, temperature, and pressure (200 °C/min., 1550 °C, 30 MPa), but with varying holding time from 0 to 30 min., smaller initial particle size resulted in higher relative density at all of the holding times. The authors concluded that the driving force for the densification of fine initial powder was greater than coarse powder during spark plasma sintering. Therefore, they used the powder with a particle size of 3.46 µm to study the effect of all other parameters. They reported that increasing the holding time increased the relative density of the sintered samples. At smaller holding times, the edge part of sintered samples was found to be more dense than the inside part, but this microstructural inhomogeneity disappeared at higher holding times. When the thickness of sintered samples was increased from 3 mm to 8 mm using constant SPS parameters (200 °C/min., 1550 °C, 10 min., 30 MPa), relative density was found to decrease from 99.5% to 97%, respectively. When the samples were sintered at different heating rates from 20 to 300 °C/min. while keeping other conditions constant (1550 °C, 10 min., 30 MPa), it was found that samples that were sintered at heating rates higher than 50 °C/min. reached 99% of theoretical densities while sample having heating rate of 20 °C/min. reached 97.6% theoretical density due to the abnormal grain growth at lower heating rates, which reduced the driving force for densification. Increasing pressure from 20 MPa to 40 MPa while keeping other parameters constant (200 °C/min., 1550 °C, 10 min.) increased the relative density from 97% to 99%.

Jinling Liu and co-workers [115] studied the influence of the particle size of the starting powder and sintering temperature on the grain refining of alumina produced by SPS. They used alumina powders with a particle size of 1µm and 3µm. They sintered the samples at different temperatures (1200 and 1300 °C), but kept other parameters unchanged at 50 MPa, 150 °C/min., 2 min. for all samples. They found that the density of the samples having smaller initial particle size i.e., 1 µm was higher and it reached 100% densification value at a temperature of 1300 °C than the sample with large initial particle size of 3 µm, which reached a value of 89%. The grain sizes of sintered samples with 1 µm initial powder size at 1200 and 1300 °C were reported as 300 nm and 2.2 µm, respectively, while for 3 µm initial powder, the grain sizes were 110 nm and 700 nm, respectively. In the case of 3 µm initial powder, regardless of sintering temperature, the final grain size of sintered samples was found to be smaller than initial powder particle size, revealing the grain refining effect that was not found in the case of 1 µm powder.

Dibyendu Chakravarty and co-workers [116] studied the effect of the addition of different sizes (100 nm, 15 nm) and different percentages of MgO (0.0625, 0.125 and 0.25%) as grain growth inhibitor during spark plasma sintering of alumina and compared it with spark plasma sintering of pure alumina. They used alumina powder having particle size of 150 nm. When the samples of pure alumina and alumina with different percentages of MgO were sintered at 1150 °C, 175 °C/min., 5 min., and 50 MPa, it was found that values of density, hardness, and fracture toughness of all the samples containing different percentages of MgO were higher than pure alumina along with less grain growth than pure alumina. The sample containing 0.125% MgO showed maximum densification along with minimum grain growth, while the hardness and fracture toughness values were also higher than others. With the increase in MgO content, it was found that some MgO segregate and remained as clusters within alumina matrix, which reduced the effectiveness of MgO as the grain growth inhibitor. Samples that were sintered with smaller particle size (15 nm) of MgO showed much higher properties with minimum grain growth than those containing larger particle size (100 nm) MgO. This was due to the fact that, in a smaller size of MgO, the amount of MgO per unit area of alumina grain boundary was large, which pinned down the grain boundaries very efficiently and restricted the grain boundary migration during sintering, resulting in finer structure.

Guo-Dong Zhan and others investigated the effectiveness of SPS for the consolidation of alumina [117]. They used an alumina nanopowder with an average particle size of 50 nm. Sintering was performed at heating rate of 200 °C/min., pressure of 63 MPa, temperature of 1150 °C, and dwell time of 3 min. A 99.8% relative density along with 349 nm average grain size of the sintered compact was obtained. This showed the effectiveness of spark plasma sintering method. A hardness value of 20.3 GPa and fracture toughness value of 3.3 MPa m^1/2^ was reported.

Maryse Demuynck and others [118] investigated the effectiveness of spark plasma sintering process by consolidating alumina powders and then compared the results with conventional hot pressing (HP). They used alumina powder with a particle size of 0.45 µm. The HP experiments were carried out under hot resistive heating conditions (HPR) and hot inductive heating conditions (HPI). Samples that were prepared form SPS and HPI were of 20 mm diameter, while those with HPR were of 30 mm diameter. It was found that the samples sintered by SPS reached much higher densities within short sintering cycles than HPR and HPI. The quick heating rates in SPS allowed for reaching the temperature range quickly where the densification process was favored and kinetically separated from grain growth. The influence of heating rate on average grain size was found to be dependent on the used technique. By increasing heating rate, no effect was observed on the average grain size of sintered samples in SPS; in HRP it led to decrease in grain size and in HPI it caused grain coarsening. In SPS, using parameters 1500 °C, 100 °C/min., 6 min. and increasing the pressure from 16 MPa to 48 MPa resulted in the increase in relative density from 65% to 92%, while the grain size also increased from 1.2 to 2.5 µm, respectively.

Alumina powder with a particle size of 200 nm was consolidated while using spark plasma sintering [119]. The powder was processed before sintering, where a slurry of alumina of concentration 30 mg/mL was formed with DMF solvent and then ball milled at 350 rpm with zirconia balls (10 mm dia.) for 4 h using powder to ball ratio (PBR) 1:20. The slurry was then dried at 90 °C for 10 h and then ground and sieved while using 250 mesh. Subsequently, further drying was done in vacuum oven at 90 °C for two days. Finally, alumina was sintered at sintering parameters of 100 °C/min., 1350 °C, 50 MPa, and 5 min. A relative density of 99.8% was achieved along with final grain size of 529 nm. Hardness, elastic modulus, and fracture toughness values reported were 22.9 GPa, 380 GPa, and 2.9 MPa m^1/2^, respectively.

Jian Liu and others [120] consolidated alumina powder with an average particle size of 150 nm by spark plasma sintering and evaluated its mechanical properties. They used 100 °C/min., 50 MPa, 1500 °C, and 3 min. as the sintering parameters. They achieved a relative density of 100%. Hardness, flexural strength, and fracture toughness values of 18.04 GPa, 400 MPa, and 3.53 MPa m^1/2^, respectively, were reported in their study.

Morales-Rodriguez et al. [121] studied the nanograin cluster coalescence and coarsening kinetics that are involved alumina sintered by SPS. They used a nanopowder having particle size that ranges between 30–40 nm. When temperature was increased from 1200 °C to 1250 °C while keeping others parameters constant (75 MPa, 10 min.), the relative density increased suddenly from 79% to 93% along with exponential grain growth from 100 nm to 290 nm. Increasing the time from 5 to 10 min. while keeping other sintering perimeters unchanged (75 MPa, 1300 °C), the relative density increased from 98% to 100% while the grain size also increased from 670 nm to 840 nm. Even when the short sintering time and low temperature were used, the nanostructure of alumina powder was lost and resulted in submicron sized grains in sintered compacts. From the XRD diffraction pattern, differences in preferred orientation of initial powder and sintered compact and smaller amount of strain were observed, which were induced during sintering and could affect mechanical characteristics. Abnormal grain growth and nanometric multi grains clusters were observed in HRSEM images. It was also found that, under different temperatures, the number of grains that form clusters inside larger grains also changes. When the nature of grain boundaries inside these clusters were studied by TEM, the results showed arrays of dislocations, which indicated that clusters consisted of subgrains separated by low angle grain boundaries. The formation of these clusters suggested that densification and grain growth during spark plasma sintering process took place mainly by grain rotation and sliding mechanism.

Guo-Dong Zhan et al. [122] used the spark plasma sintering technique to consolidate alumina powder having a particle size of 50 nm and evaluated its mechanical properties. The authors used a heating rate of 200 °C/min., pressure of 63 MPa, temperature of 1150 °C, and time of three min. A relative density of 99.8% along with the grain size of 349 nm was reported. Fracture toughness was found to be 3.3 MPa m^1/2^.

The effect of improper selection of sintering parameters on the sintering of alumina powder having a particle size of 0.21 µm was investigated [123]. When pressure was increased from a very low value of 5.5 MPa to higher pressure of 15 MPa while other parameters were kept constant (200 °C/min., 1250 °C, 5 min.), the relative density increased from 93% to 99% and the grain size decreased from 0.67 µm to 0.58 µm. It was found that the specimens compacted using lower pressure of 5.5 MPa contained inhomogeneous microstructure, while those that were sintered at high pressure of 15 MPa contained fine microstructure. An enhanced densification rate and homogeneous microstructure at higher pressure were thought to be due to the fact that under higher pressure; each particle was surrounded by increased number of particles. Hardness, elastic modulus, and strength values were also increased due to the decrease in grain growth reaching values of 19.5 GPa, 380 GPa and 741 MPa, respectively, at 15 MPa. Fracture toughness was found to decrease from 4.4 to 2.2 MPa m^1/2^. When the heating rate was increased from 100 °C/min. to 200 °C/min. while other parameters were kept constant (1250 °C, 15 MPa, 5 min.), the relative density increased from 98% to 99% and the grain size decreased from 0.68 µm to 0.58 µm. A homogenous microstructure was observed in both cases. Higher hardness, strength, and modulus values were reported at higher heating rate of 200 °C/min. due to a decrease in grain size, but fracture toughness value was reduced.

You Zhou et al. [124] studied the densification and grain growth behavior during spark plasma sintering of alumina powder. Alumina powder with a particle size of 0.15 µm was used. The holding time was kept at 0 min. and pressure at 47 MPa in all experiments. At 1000 °C, when the heating rate was increased from very low to very high i.e., 50 °C/min. to 300 °C/min., the relative density slightly increased from 61.2% to 63.5%, but no grain growth was observed. Different microstructures were observed in SEM images. In the case of very fast heated specimen at 300 °C/min., necking extensively occurred between the neighboring particles, while in the case of slow heated specimen at 50 °C/min., no such phenomenon was observed. No grain growth was observed up to the temperature of 1100 °C, irrespective of heating rate, while density increased as temperature and heating rate was increased. When the temperature increased above 1150 °C, grain growth started, along with increased densification. At very high temperature i.e., 1400 °C, when heating rate increased from very low at 50 °C/min. to very high 300 °C/min., the density slightly decreased from 99.4% to 99.3%, while grain size decreased from 7.67 µm to 2.37 µm. Three stages were identified during spark plasma sintering: densification without grain growth, densification along with grain growth, and grain growth without further densification.

Morales-Rodriguez and others [125] used spark plasma sintering for the consolidation of alumina powder with a particle size 30–40 nm. A heating rate of 300 °C/min., pressure of 75 MPa, temperature of 1300 °C, and time of 5 min. were used. The authors reported a relative density of 97.7%, grain size of 700 nm, and hardness value of 19 GPa.

Shan Meng and co-workers investigated the effect of sintering temperature and sintering aid content on densification and grain growth of spark plasma sintered alumina powder having an average particle size of 150 nm [126]. They used different percentages of MgO (0–0.4 wt%) as grain growth inhibitors. They found that the highest densification along with minimum grain growth was obtained with 0.05 wt% MgO. Accordingly, 0.05 wt% MgO doped alumina was used for further study. A highest density of 99.8% along with minimum grain size of 680 nm was obtained when sintering was performed at 1250 °C, 100 °C/min., 60 MPa, and 5 min. The hardness and fracture toughness values were found to be 20.75 GPa and 4.45 MPa m^1/2^, respectively. When the temperature was increased from 1250 °C to 1500 °C, a slight increase in density was observed, but grain sizes increased exponentially to 4 µm. Hardness and fracture toughness values were also decreased due to huge increase in grain size.

Kasperski and co-workers [127] used the spark plasma sintering process for the consolidation of alumina powder with particle size of 140 nm. When alumina was sintered at higher temperature and higher pressure (1350 °C, 150 MPa) at heating rate of 100 °C/min. and for 6 min., 99% relative density was obtained along with grain size of 1.2 µm. The hardness and fracture toughness values were found to be 22 GPa and 5 MPa m^1/2^, respectively. When temperature, pressure and time were reduced to 1150 °C, 100 MPa and 5 min., relative density increased to 100% while the grain size was reduced to 320 nm. The hardness and fracture toughness values were found to be 21.3 GPa and 5.4 MPa m^1/2^, respectively.

Ken Hirota et al. [128] used spark plasma sintering to compact alumina powder with a particle size of 300 nm. They used a heating rate of 100 °C/min., pressure of 30 MPa, temperature of 1300 °C, and time of 5 min. They reported a relative density of 99.3% and grain size of 4.4 µm. In addition to hardness and fracture toughness values of 19.5 GPa and 4.7 MPa m^1/2^, respectively.

Alvarez-Clemares and co-workers [129] studied the effects of spark plasma sintering on microstructure, mechanical properties, and creep behavior of alumina ceramic. They used alumina powder with an average particle size of 153 nm. A heating rate of 50 °C/min., pressure 80 MPa, temperature 1300 °C and time 2 min. were used for sintering. A 99.9% relative density was achieved. It was found that some amount of strains were induced in samples during sintering, so, after sintering, some samples were annealed to 1000 °C for 5 h to eliminate the residual stresses. Creep deformation for as-sintered specimen was found to be 1.4%, while 22% for annealed specimen. This was due to the fact that, during the sintering process, dislocations were induced that blocked the grain boundary movement, which resulted in low deformation of as-sintered specimen. Fracture toughness and flexural strength for as-sintered specimen were found to be 4.3 MPa m^1/2^ and 430 MPa, respectively, and 3.4 MPa m^1/2^ and 275 MPa, respectively, for the annealed specimen. The authors concluded that SPS induced strains that concentrate at grain boundaries and inhibit the crack growth, resulting in improved fracture toughness and flexural strength of the as-sintered specimen. They confirmed that SPS process induces strains that affect the mechanical properties of the sintered specimen.

**Figure 8:**
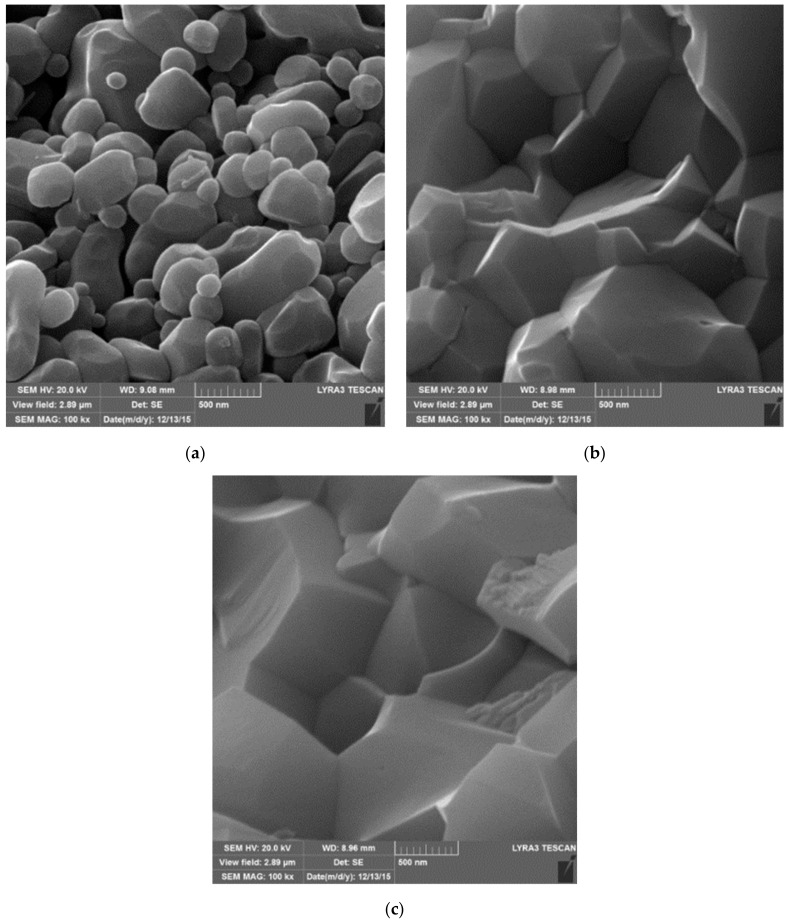
FE-SEM images of fractured surfaces of alumina samples sintered for 10 min. at (**a**) 1000 °C, (**b**) 1300 °C, and (**c**) 1400 °C [132].

**Figure 9:**
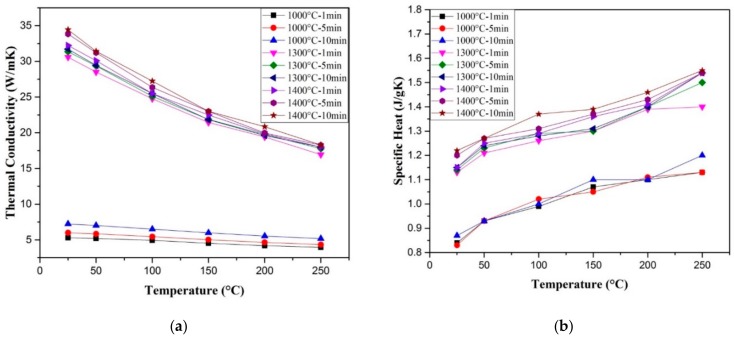
(**a**) Thermal conductivity and (**b**) specific heat of sintered alumina [132].

Nanostructured alumina was consolidated to full density by SPS at a sintering temperature of 1150 °C and sintering time of 3 min. [130]. The authors reported a value of 27.5 W/mK for the room temperature thermal conductivity of the fully dense alumina. In addition, they found that the increase in temperature from 25 °C to 500 °C leads to a decrease in thermal diffusivity from 0.088 to 0.03 cm^2^/sec. In another work, the thermal conductivity of nanostructured alumina (200 nm), spark plasma sintered at 1400 for 3 min. (50 MPa), was found to decrease from around 34 to 13 W/mK with the increase in temperature from 300 to 800 K [131]. An almost fully dense monolithic Al_2_O_3_ (99.6% of the theoretical density), as can be seen in Figure 8, was consolidated at 1400 °C for 10 min. The thermal conductivity and specific heat were about 34.44 W/mK and 1.22 J/gK, respectively, at room temperature. Thermal conductivity was found to decrease with the increase in temperature and it reached 18.3 W/mK at 250 °C, while specific heat increased and reached a value of 1.55 J/gK at 250 °C, as shown in Figure 9 [132].

## 8. Spark Plasma Sintered Alumina Hybrid Nanocomposites

The development of alumina hybrid nanocomposites using the spark plasma sintered method is in its early stage and only limited research work is available in the literature. However, the reported results are very promising and potential applications are amazing. Analysis of the literature shows that diverse nanoreinforcements were used to prepare alumina hybrid nanocomposite materials. This includes Al_2_O_3_-SiC-CNTs [32,40,41,43,133], Alumina-GNPs-SiC [39,134], Alumina-Graphene-CNTs [53,135], Alumina-modified multilayer graphene nanoplatelets [136], Al_2_O_3_–ZrO_2_-Graphene [137], Al_2_O_3_-SiCw-TiC [54,55], Al_2_O_3_-TiC-Ni [56], Al_2_O_3_-CNFs-SiC [57], and Alumina-Graphene based nanocomposites [58,59]. The majority of the performed studies were dedicated to the evaluation of mechanical properties and few researchers considered the tribological [54], electrical [40,54,58,59,60], and thermal properties [40,60].

### 8.1. Al_2_O_3_-SiC-CNTs

Almost fully dense Al_2_O_3_-SiC-CNTs composites (higher than 98%) were obtained by SPS at 1500 °C for 10 min. while using a pressure of 50 MPa [41]. Moderate relative density values of at least 95.1% [32] and 96.4% [40] were reported for Al_2_O_3_-SiC-CNT and Al_2_O_3_-CNT-SiC composites, respectively, produced by SPS at 1550 °C and 50 MPs. In addition, a relatively low relative density value of 91.65% was reported for Al_2_O_3_-SiC-CNTs that were synthesised by MLM and consolidated by SPS at 50 MPa, 1500 °C, and 10 min. [43].

**Figure 10:**
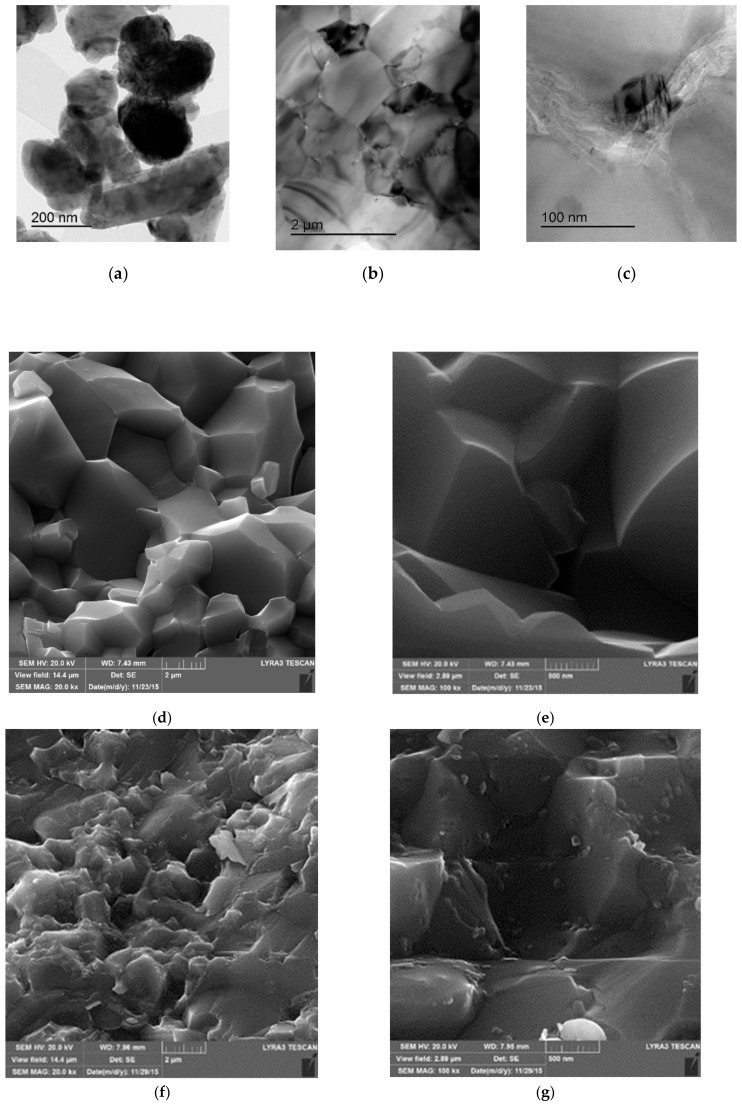

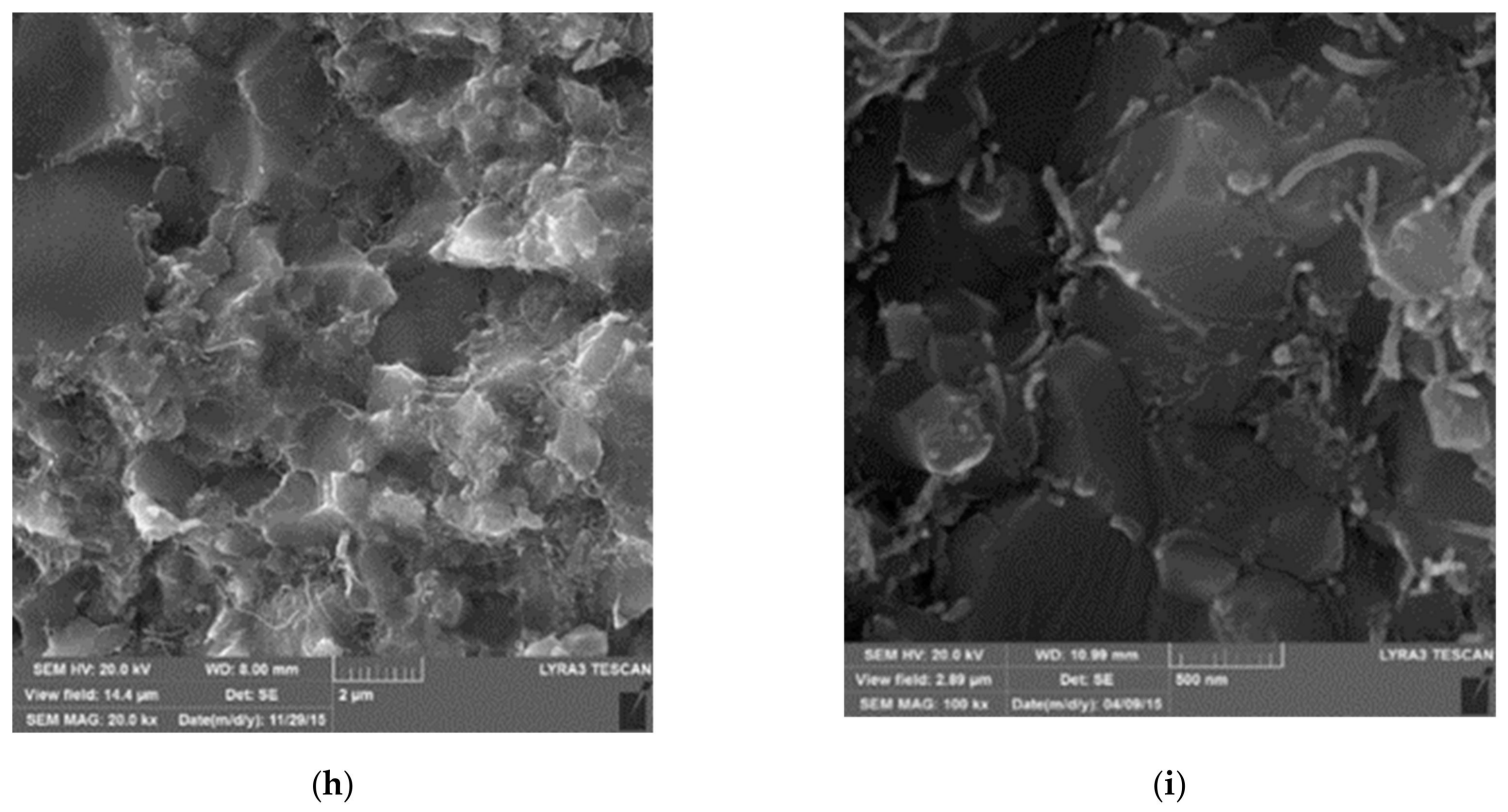
(**a**) TEM image of Al_2_O_3_ powder, (**b**,**c**) TEM images of sintered Al_2_O_3_-5SiC-1CNT, Fracture surfaces of (**d**,**e**) Al_2_O_3_, (**f**,**g**) Al_2_O_3_-10-SiC, and (**h**,**i**) Al_2_O_3_-10SiC-2CNTs (Reproduced with permission from [41]. Elsevier, 2016).

It was found that the average grain size of the alumina matrix in Al_2_O_3_-5SiC-1CNTs hybrid composite did not significantly change [41]. This can be seen from a typical TEM image of the composite that is presented in Figure 10b, as compared with the average particle size of the alumina starting powder presented in Figure 10a. This is believed to be due to the pining effect of CNTs and SiC that reside on grain boundaries, as indicated by dark and white arrows, respectively, as in Figure 10c.

Figure 10d–i present typical FE-SEM images of fracture surfaces of monolithic alumina Al_2_O_3_, Al_2_O_3_-10-SiC composite, and Al_2_O_3_-10SiC-2CNTs hybrid composite [41]. The SiC nanoparticles are present inside the grains and on the grain boundaries, as can be seen in Figure 10h. The comparison of the microstructures of the monolithic alumina and the Al_2_O_3_-10-SiC composite presented in Figure 10d,e, respectively, reveals the role of SiC nanoparticles in restricting the growth of the alumina grains. Figure 10i shows that CNTs remained uniformly distributed in the sintered hybrid composite.

**Figure 11:**
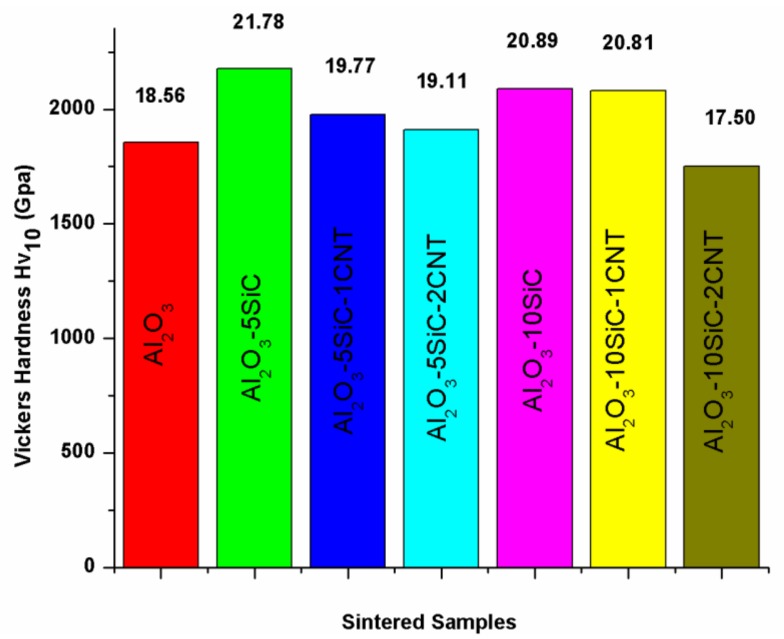
Hardness of Al_2_O_3_-SiC-CNTs hybrid composites prepared by SPS (Reproduced with permission from [41]. Elsevier, 2016).

Saheb and Khwaja [41] investigated the effect of 0D SiC nanoparticles and 1D CNTs on the hardness of Al_2_O_3_-SiC-CNTs. They found that the hybrid nanocomposites, except Al_2_O_3_-10SiC-2CNTs, had improved the hardness with respect to Al_2_O_3_. Among the hybrid nanocomposites, Al_2_O_3_-10SiC-1CNTs had the highest hardness value of 20.81, as can be seen in Figure 11. The improvement in the hardness was attributed to the refinement of the Al_2_O_3_ matrix, as can be observed on the fracture surfaces that are presented in Figure 10, and the hardness of SiC particles.

Researchers who investigated the effect of SiC nanoparticles (1, 2, 3 vol%) on the hardness of Al_2_O_3_-5vol% CNTs [40] and CNTs (0, 5, 7, and 10 vol%) on the hardness of Al_2_O_3_-1vol%SiC reported hardness values that range from 14 and 17 GPa [32]. The nanocomposites displayed lower hardness compared to the pristine alumina (17 GPa). In another work, the Al_2_O_3_-5SiC-1CNTs composite that was synthesised by MLM [43] was found to have a reduced hardness by ∼4% with respect to monolithic Al_2_O_3_.

The influence of SiC (1, 2, 3 vol%) on the flexural strength of Al_2_O_3_-5vol% CNTs [40] and CNTs (0, 5, 7, and 10 vol%) on the flexural strength of Al_2_O_3_-1vol%SiC [32] was investigated. The authors reported flexural strength up to 500 MPa for the hybrid composites as compared to 330 MPa for monolithic alumina.

**Figure 12:**
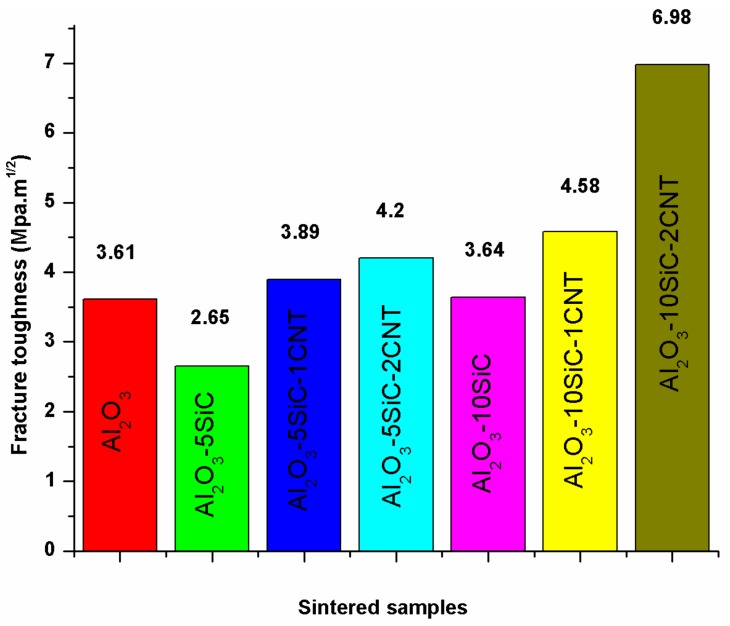
Fracture toughness of Al_2_O_3_-SiC-CNTs hybrid nanocomposites prepared by SPS (Reproduced with permission from [41]. Elsevier, 2016).

Saheb and Khwaja [41], in their investigation of the influence of CNTs (1 and 2 wt.%) on the fracture toughness of Al_2_O_3_-SiC (5 and 10 wt.%) nanocomposites, found that the Al_2_O_3_-SiC-CNTs hybrid nanocomposites had improved fracture toughness when compared to monolithic alumina, as can be seen in Figure 12. The composites demonstrated improved fracture toughness with respect to Al_2_O_3_. A highest fracture toughness value of 6.98 MPa m^1/2^ was reported for the Al_2_O_3_-10SiC-2CNTs composite as compared to a value of 3.61 MPa m^1/2^ for alumina i.e., an increase of 93.95%. This increase was attributed to the change of fracture mode from intergranular to a mixture of intergranular and transgranular modes, because of SiC addition, and to a complete transgranular fracture mode due to the addition of both SiC and CNTs.

**Figure 13:**
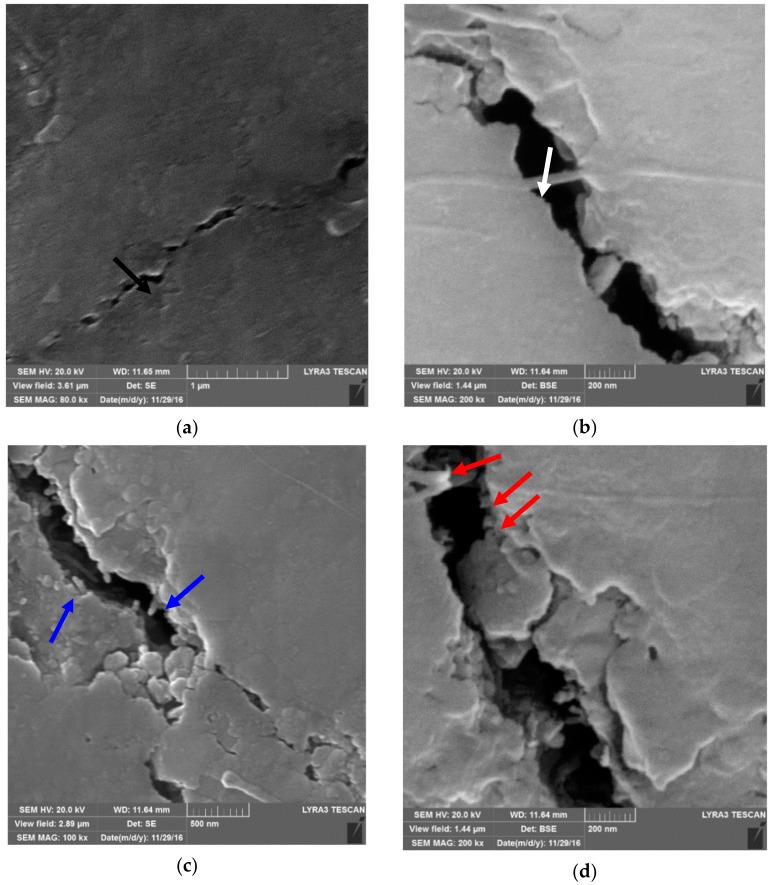
FE-SEM images of Al_2_O_3_-5SiC-1CNT nanocomposite, sintered at 1600 °C, revealing toughening mechanisms (**a**) crack deflection (**b**) crack bridging, (**c**) CNT breaking and (**d**) CNT pullout (Reproduced with permission from [133]. Springer Nature, 2017).

In another study that was performed by Khwaja and Saheb [43], a fracture toughness value of 5.83 MPa m^1/2^ was reported for the Al_2_O_3_-5SiC-1CNTs composite, synthesised by molecular level mixing, as compared to a value of 3.61 MPa m^1/2^ for Al_2_O_3_ i.e., an increase of 33%. The relative density of this composite was 91.65%. The same authors [133] found that, for Al_2_O_3_-5SiC-1CNT nanocomposite, the increase in sintering temperature from 1500 °C to 1600 °C led to the increase of the relative density from 90.36% to 98.91%. They reported hardness and fracture toughness values of 23.32 GPa and 7.10 MPa m^1/2^, respectively, for the sample that was sintered at 1600 °C for 10 min. This represents an increase in the hardness and fracture toughness of 25.65 and 96.67%, respectively, when compared to monolithic alumina sintered at 1500 °C for 10 min. The authors attributed “the concurrent increase in hardness and fracture toughness to the uniform distribution of the reinforcements, to high densification, to the refinement of the alumina matrix grain size, to a change in fracture mode, and to the toughening mechanisms that are brought about by the SiC nanoparticles and the CNTs”, as shown in Figure 13.

Other researcher investigated the effect of SiC (1, 2, 3 vol%) on the fracture toughness of Al_2_O_3_-5vol% CNTs [40] and CNTs (0, 5, 7, and 10 vol%) on the fracture toughness of Al_2_O_3_-1vol%SiC [32] and reported values of up to 6 MPa m^1/2^. The authors concluded that the increase in fracture toughness was due to grain boundaries strengthening and toughening of the alumina matrix brought about by SiC nanoparticles and MWCNTs. “The incorporation of SiC nanoparticles is also believed to remove residual stresses at the alumina-alumina boundaries, and in matrix grains by generating dislocations around the particles [138,139]. The elimination of tensile stresses strengthens the grain boundaries and it impedes the intergranular fracture that was observed in alumina with added CNTs”.

**Figure 14:**
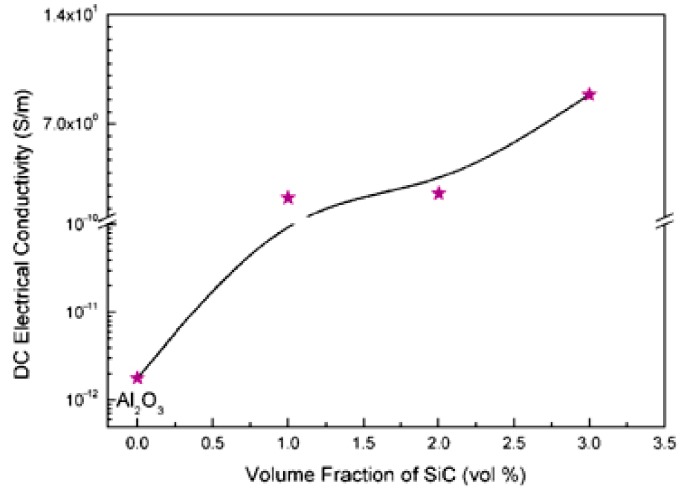
Variation of electrical conductivity of alumina and hybrid nanocomposites with volume fraction of SiC at room temperature (Reproduced with permission from [40]. John Wiley and Sons, 2009).

The incorporation of hybrid nanoreinforcements in alumina significantly increased its electrical conductivity. A high value of ≈9 S/m was obtained by the addition of 3 vol% of SiC and 5 vol% of CNTs to alumina, as can be observed in Figure 14 [40]. The increase was believed to be mainly due to networking of CNTs along alumina grain boundaries, despite the fact that SiC is known to contribute to the enhancement of the electrical conductivity.

A high electrical conductivity value of 8.85 S/m was reported for the Al_2_O_3_-5SiC-2CNTs nanocomposite as compared to a low value of 6.87 × 10^−10^ S/m for the pristine alumina. This makes the composite material suitable for electrical discharge machining (EDM), regardless its hardness or strength, and it enables the production of cheap and complicated components and parts [60].

**Figure 15:**
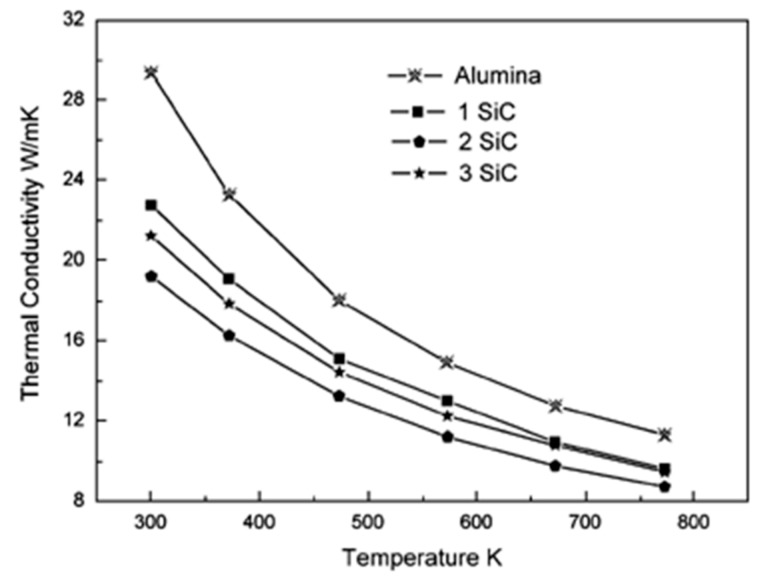
The dependence of the thermal conductivity of spark plasma sintered alumina hybrid ceramic nanocomposites on temperature (Reproduced with permission from [40]. John Wiley and Sons, 2009).

Other researchers [40] investigated the thermal conductivity of Al_2_O_3_-SiC-CNTs hybrid nanocomposites reinforced with CNTs (5 vol%) and SiC (1, 2, 3 vol%). They found it to decrease for 1 and 2 vol% of SiC and then increase for 3 vol%. However, it remained lower than that of pure Al_2_O_3_, as can be seen in Figure 15. The authors used the specific heat of graphite for CNTs [140,141] to calculate the specific heat of the nanocomposite materials while using the rule of mixture.

### 8.2. Alumina-SiC-Graphene

**Figure 16:**
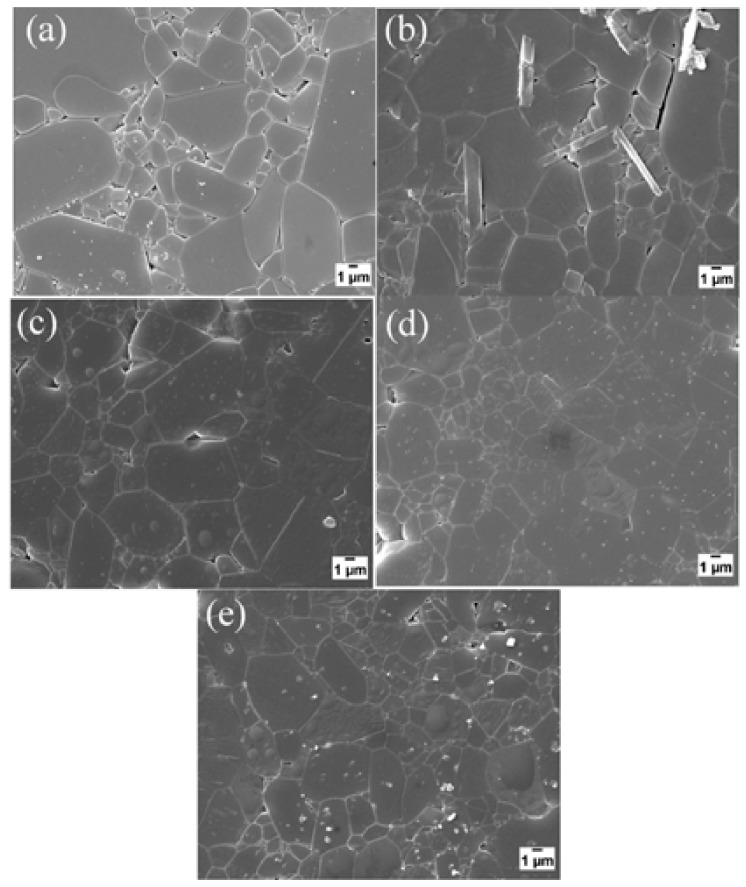
SEM images of thermally etched surfaces of sintered pure Al_2_O_3_ (**a**), GPL-Al_2_O_3_ composite (**b**), GPL/1 vol% SiC-Al_2_O_3_ composite (**c**), GPL/3 vol% SiC-Al_2_O_3_ composite, (**d**) and GPL/5 vol% SiC-Al_2_O_3_ composite (**e**) (Reproduced with permission from [39]. John Wiley and Sons, 2014).

Alumina reinforced with graphene platelets (GNPs) and silicon carbide (SiC) hybrid nanocomposites with high relative density values of at least 97.35% were produced by SPS (1500 °C, 3 min., and 50 MPa) [39]. The composites had uniform and refined microstructures, as can be seen from thermally etched surfaces of sintered samples that are presented in Figure 16 [39]. The refinement of the microstructure was credited to grain boundary pinning by GNPs and SiC. The addition of 0.38 vol.% of GNPs to Al_2_O_3_-SiC (1, 3, 5 vol.% SiC) was found to increase the hardness from 18.04 to 24.65 GPa, and this increase was accompanied with a slight decrease in densification. In addition, the flexural strength and fracture toughness increased to maximum values of 572 MPa 5.03 MPa m^1/2^, respectively. The increase of fracture toughness was attributed to the good dispersion of GNPs bought about by the presence of the hard SiC nanoparticles, GNP pullout, and crack bridging and deflection mechanisms that are imparted by GNPs.

**Figure 17:**
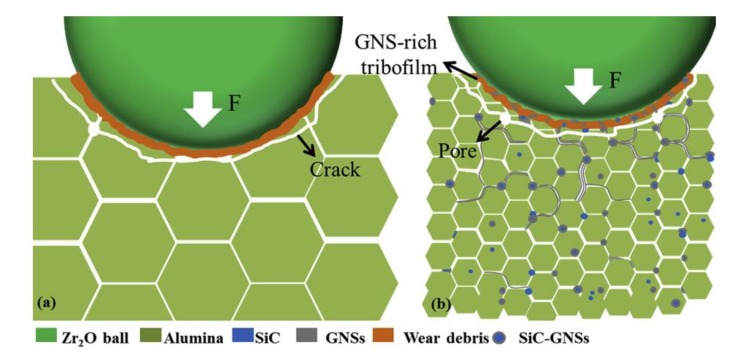
Schematic of the wear behaviour of Al_2_O_3_ (**a**) and (**b**) Al_2_O_3_-SiC-GNSs composites (Reproduced with permission from [134]. Elsevier, 2019).

Homogenous alumina hybrid nanocomposites that were reinforced by SiC-graphene nanosheets (GNSs) core-shell nanoparticles were prepared while using wet ball milling, ultra-sonicaton, and spark plasma sintering methods [134]. The well dispersed SiC-GNSs nanofillers, in the Al_2_O_3_ matrix, led to small grain sizes, increased hardness, moderate improvement in fracture toughness, reduced coefficient of friction, and significant improvement in the electrical conductivity. The authors of this work concluded that the combined effect of GNSs-rich tribofilms, as illustrated in Figure 17, refined microstructure, improved toughness and hardness, as shown in Table 1, contributed to the improvement of the wear-resistant, and self-lubricating performance of the developed hybrid nanocomposites.

### 8.3. Alumina-Graphene-CNTs

**Figure 18:**
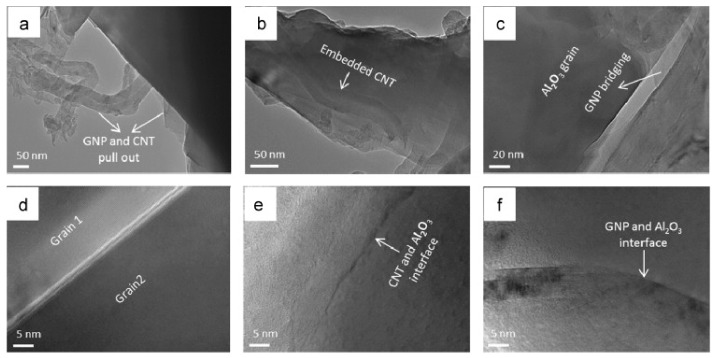
(**a**–**f**) TEM images of spark plasma sintered Al_2_O_3_-0.5CNTs-0.5GNPs composite (Reproduced with permission from [53]. Elsevier, 2015).

At least 98% dense Al_2_O_3_-graphene-CNT hybrid nanocomposites [53] were produced by SPS at 40 MPa, and 1650 °C for 10 min. Their hardness values were found to increase from 13.5 GPa (monolithic alumina) up to 15.5 GPa (Al_2_O_3_-0.5wt.GNT-1wt.%CNT), and then decrease to 11.2 GPa (Al_2_O_3_-1wt.GNT-1wt.%CNT). The flexural strength increased from 350 MPa (Al_2_O_3_) to a value of 450 MPa for (Al_2_O_3_-0.5wt.GNT-0.5wt.%CNT), and then decreased to a value of 325 MPa for (Al_2_O_3_-0.5wt.GNT-1wt.%CNT). The fracture toughness increased up to a value of 5.75 MPa m^1/2^ (Al_2_O_3_-0.5GNPs-0.5CNTs). The improvement in fracture toughness was attributed to the following: (i) anchoring around of alumina grains by GNPs, (ii) interface friction between the reinforcement and matrix, (iii) GNP pull out, (iv) change of fracture mode to transgranular, and (vi) crack bridging by GNPs and CNTs. Figure 18 shows the TEM images of alumina containing 0.5% GNPs and 0.5% CNTs. GNP pullout, embedded CNT, and GNP bridging can be clearly seen in Figure 18a–c, respectively. In addition, the firmly attached CNTs and GNPs to the Al_2_O_3_ grain, as observed in Figure 18e,f, respectively, indicate strong interface between the matrix and reinforcement. In another work [121], the addition of GNPs to Al_2_O_3_ was reported to increase the fracture toughness and decrease the hardness.

**Figure 19:**
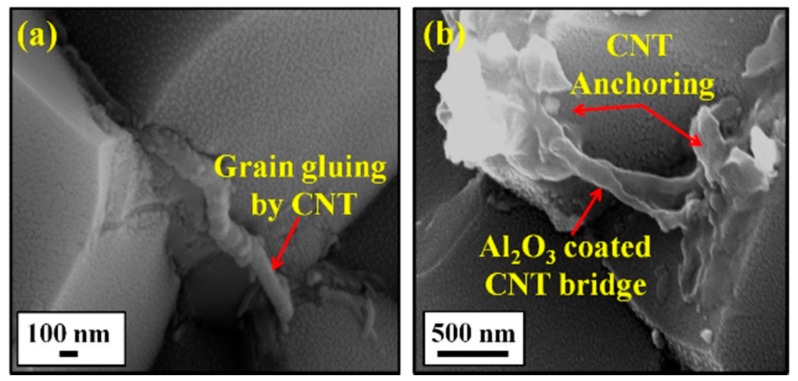
(**a**) CNT-grain gluing and (**b**) CNT-grain bridging or anchoring in the composite (Reproduced with permission from [135]. Elsevier, 2018).

**Figure 20:**
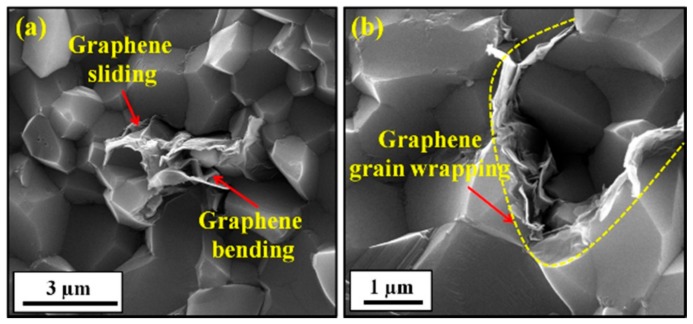
(**a**) Graphene sliding and bending between Al_2_O_3_ grains and (**b**) graphene-grain wrapping (Reproduced with permission from [135]. Elsevier, 2018).

**Figure 21:**
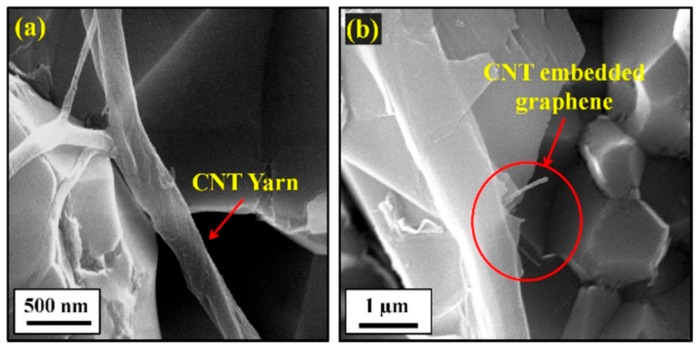
(**a**) CNT yarn in Al_2_O_3_-1wt% CNT and (**b**) CNT implanted graphene in Al_2_O_3_-1wt% CNT-0.5 wt% GNP sintered nanocomposites (Reproduced with permission from [135]. Elsevier, 2018).

Asiq Rahman and co-workers [135] prepared monolithic alumina, CNT (1 wt.%) reinforced alumina nanocomposite, and alumina hybrid nanocomposite reinforced with 1 wt.% CNTs and 0.5 wt% Graphene Nanoplatelets (GNP). The samples were consolidated by SPS at 1500 °C. The hybrid nanocomposite showed a higher density than the monolithic alumina and alumina nanocomposite. This is believed to be due to combination of very high thermal and electrical conductivity of GNPs (5000 W/m K and ∼108 S/cm) and higher contact area of GNPs with the Al_2_O_3_ particles. The high contact area facilitates the transfer of large amount of heat to alumina grains and it enhances surface melting, which could lead to the formation of grain necking and it significantly enhances the diffusion rate that finally leads to improved densification. Grain boundary pinning by CNTs and grain wrapping by GNPs were found to be responsible for the refinement of the microstructure of alumina matrix. A clear interface was seen between Al_2_O_3_, CNT, and GNP and no additional phases were formed. The hybrid nanocomposite showed increased interfacial shear stress, which was attributed to the relatively stronger and larger interfacial interaction of graphene sheet with the matrix. The addition of both CNTs and GNPs to alumina led a substantial improvement of 250% in the fracture toughness and high increase in the critical energy release rate, a measure of the amount of energy that is required for crack propagation. The drastic improvement in toughness was attributed to not only the traditional toughening mechanisms, such as CNT pullout, grain gluing, bridging and graphene pullout, bending, sliding, and grain wrapping, as it is clear from Figure 19 and Figure 20, but also to novel toughening mechanisms, such as CNT yarning and CNT embedded graphene, as shown in Figure 21. Additionally, the increased interfacial shear stress might have contributed to the improvement of toughness.

### 8.4. Alumina-Modified Multilayer Graphene Nanoplatelets

**Figure 22:**
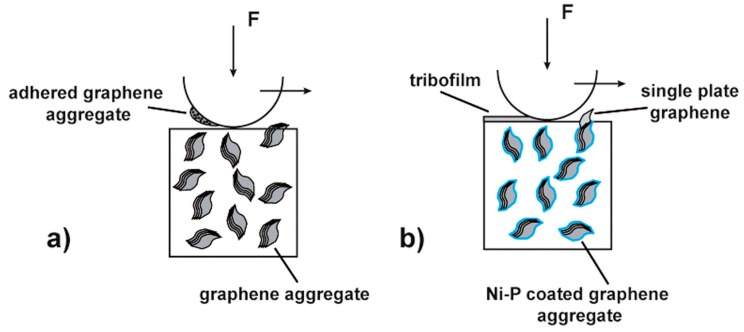
Schematics of the self-lubricating tribofilm formation in the MLG (**a**) and MLG-Ni-P (**b**) reinforced composites (Reproduced with permission from [136]. Elsevier, 2018).

Wozniak et al. investigated the effect of modified multilayer graphene nanoplatelets (MLG-Ni-P) and unmodified multilayer graphene nanoplatelets (MLG) addition on the relative density, fracture toughness, tribological behavior, and strength properties of spark plasma sintered alumina matrix composites [136]. The authors reported that the addition of graphene that was coated by metallic layer to alumina improved its density and mechanical properties. However, the tribological properties were not improved as expected. A lubricating tribofilm was observed for Al_2_O_3_-1vol% MLG-N-P composites, as can be seen in Figure 22b, but the film was quickly damaged due to its small thickness. For composites reinforced with uncoated MLG, which displayed better tribological properties, the lubricating layer was found to form on the worn surface of the ball and not on the composite, as shown in Figure 22a. The authors concluded that at least 1 vol% graphene is necessary for significantly improving the wear resistance.

### 8.5. Al_2_O_3_–ZrO_2_-Graphene

Petrus et al. [137] investigated the influence of multilayer graphene (MLG), graphene oxide (GO), and nickel-coated graphene (Gn-Ni-P) addition on the relative density, microstructure, hardness, and fracture toughness of Al_2_O_3_–ZrO_2_ and Al_2_O_3_–Ti(C,N) nanocomposites. The authors found that it is possible to improve the hardness and wear resistance of these composites. However, the fracture toughness of alumina remained unchanged with the addition of GO and decreased due to the addition of both MLG and Gn-Ni-P. This change in mechanical properties was attributed to the differences in the quality of the interface or its absence and the presence of additional phases of the matrix material (ZrO_2_ and Ti(CN) particles). The wear rate of the nanocomposite containing 0.2wt% GO increased by almost 800%. Other composites also showed an increase in wear resistance, depending on the type of matrix, the type of reinforcements, and the applied load.

### 8.6. Al_2_O_3_-SiCw-TiC

It was reported that fracture toughness of alumina could be significantly increased by the addition of titanium carbide [142,143]. In fact, the reinforcement of Al_2_O_3_-SiCw with 22vol.% of TiC [54,55] improved the densification, and increased the hardness, flexural strength, and fracture toughness to 22.74 GPA, 648 MPa, and 6.5 MPa m^1/2^, respectively.

### 8.7. Al_2_O_3_-TiC-Ni

**Figure 23:**
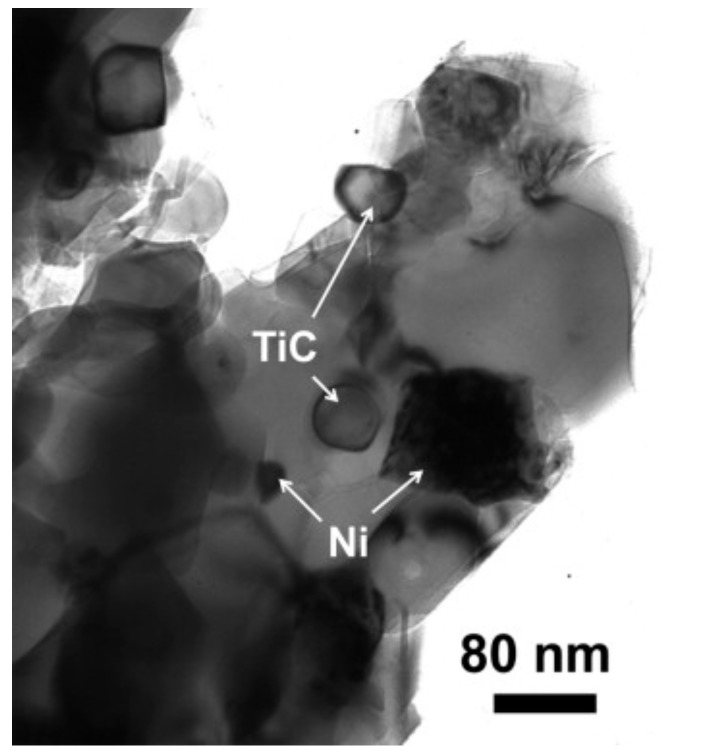
TEM micrograph of spark plasma sintered Al_2_O_3_-TiC-Ni nanocomposite (Reproduced with permission from [56]. Elsevier, 2011).

An almost fully dense Alumina-TiC-Ni nanocomposite, with relative density as high as 98%, was produced by SPS [56]. Figure 23 presents a typical TEM micrograph showing the presence of Ni and TiC nanoparticles and the limited grain growth of the alumina matrix. While the densification remained unchanged, the hardness increased by approximately 30% from 19.9 GPa for pure alumina to 25.6 GPa for the Al_2_O_3_-1.9vol.% nNi-25 vol.% nTiC nanocomposite. This increase was attributed to Ni and TiC nanoparticles/’ hardening effects. The flexural strength increased from 395 to 537 MPa; and, the fracture toughness slightly changed from 3.5 to 3.7 MPa m^1/2^. The electrical resistivity value of the alumina-TiC-Ni hybrid nanocomposite was found to be around 3.15 × 10^−5^ Ω.m, much lower than the limit of 1–3 Ω.m needed for EDM machining [56].

### 8.8. Al_2_O_3_-CNFs-SiC

The addition of carbon fibres and SiC to alumina was reported to decrease the fracture toughness of alumina to a value as low as 2.79 MPa m^1/2^ for the Al_2_O_3_-20vol.%CNF-10vol.SiC hybrid nanocomposite [57].

### 8.9. Modelling of Spark Plasma Sintered Alumina Hybrid Nanocomposites

Siddiqui et al. presented an improved model for estimating the thermal conductivity of nanocomposites prepared by SPS [144]. In the developed model, the thermal conductivity of the matrix was modeled as a function of the average matrix crystallite size rather than taking a constant matrix thermal conductivity, as in Equation (22).

(22) $$K_{pc}=\frac{34.55[W/m\cdot K]}{1+{\left(\frac{\left(2.195\times 10^{-9}[m^{2}\cdot K/W]\right)\left(34.55[W/m\cdot K]\right)}{d[m]}\right)}^{8.814}}$$

**Figure 24:**
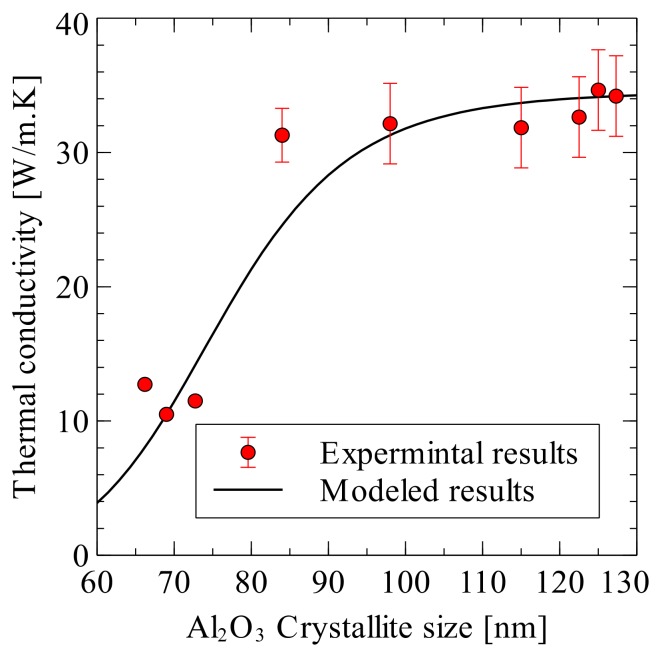
Predicted and measured thermal conductivity of alumina as a function of its average crystallite size [144].

Figure 24 shows the predicted alumina thermal conductivity as a function of the average crystallite size plotted against the experimentally measured values.

The model has been validated against experimentally measured thermal conductivity of Al_2_O_3_-SiC-CNT hybrid nanocomposites. It was shown that the addition of SiC and CNT inclusions to alumina resulted in a decrease in its thermal conductivity while using the experimental and modeling results, as seen in Table 2 [144]. The main reason for this decrease was found to be the reduction in the thermal conductivity of alumina matrix itself because of the reduction in crystallite size. Additional reduction in the composite thermal conductivity was due to the matrix-inclusion interface resistance and porosity.

### 8.10. Overall Change in Properties

The use of SPS allowed for the sintering of alumina hybrid nanocomposites, for short periods at low temperatures, to either near-theoretical density or high relative density values. The selection of SPS parameters has led to not only high relative density values, but also small grain sizes of the Al_2_O_3_ matrix [53]. Additionally, the presence of reinforcements was found to restrict the growth of alumina grains due to the pining effect. The high densification of alumina hybrid nanocomposites prepared by SPS was attributed to: (i) the high tendency of nanoparticles to sinter, (ii) promoted diffusion mechanisms, (iii) contribution of temperature, pressure, and current. Additionally, the strengthening of alumina by conducting or semi conducting phases was also found to improve the densification [54,55,56].

The use of hybrid reinforcements was found to increase the hardness of alumina [25,27,53,54,55,56], except in few studies [17,26,31] where the hardness was reported to decrease. The increase in the hardness of alumina hybrid nanocomposite materials was believed to be due to the (i) the high hardness of reinforcements, such as SiC and TiC, (ii) the refinement of the matrix due to the pinning effect that was brought about by the reinforcements, and (iii) high densification. However, the hardness might decrease because of reduced densification and the presence of inter-granular soft phases, such as CNTs, which have hardness values from 6 to 10 GPa in the radial direction [145]. Moreover, the weak interface between the reinforcement and Al_2_O_3_, the lubricating nature of some nanophases, such as CNTs and graphene, and agglomeration [30] may offset the influence of microstructure refinement [146,147,148] and the advantages of the SPS process. It is worth mentioning here the sensitivity of hardness to testing load [149] and residual internal stresses [150] that are caused by the thermal mismatch between alumina and the reinforcement.

The use of hybrid reinforcements was found to increase the strength of alumina [17,25,26,53,54,56], except in the case of carbon fibres and SiC [57], which were reported to decrease the strength of Al_2_O_3_-20vol.%CNF-10vol.SiC composite to a value as low as 144 MPa. As for toughness, the addition of hybrid reinforcements to alumina led to either a significant increase [17,25,26,27,31,53,55] or marginal improvement [56], but a decrease [57] in the fracture toughness was also reported. The discrepancy in the reported values of fracture toughness could be attributed to the: (i) type, attributes, volume, and dispersion of the reinforcements, (ii) densification, (iii) strength of interface between the reinforcement and matrix, (iv) operating toughening mechanisms, and (v) accuracy of testing methods.

Table 3 presents a summary of the processing conditions, microstructure features, type and percentage of reinforcements, and mechanical properties of some spark plasma sintered alumina hybrid nanocomposites.

## 9. Potential Applications

The hybrid microstructure design that is achieved by incorporating nanoscale phases, namely nanoparticles, nanotubes, and nanoplates into alumina, has led to novel properties that are capable of meeting or even exceeding design expectations. The augmentation in the mechanical properties of alumina hybrid nanocomposites are expected to lead to not only enhanced performance of these materials in traditional applications, such as cutting tools [4], dental implants and chemical and electrical insulators [5], and armouries [6], but also novel applications. Nanocomposites that showed increased hardness, flexural strength, and fracture toughness [25] are potential materials for structural applications. Other nanocomposites displayed a significant improvement in the electrical conductivity that made them electrically conductive ceramics i.e., they have electrical resistivity lower than 1–3 Ω.m [58]. This allows for the manufacturing of low cost products that have intricate shapes by EDM, irrespective of their hardness or strength [54,58]. These electrically conductive alumina hybrid composites could be widely applied where “high electrical conductivity is required, such as heating elements, electrical igniters, antistatic, and electromagnetic shielding effectiveness of electronic components” [56]. The improved toughness and machinability, as well as tribological properties of the recently developed novel Al_2_O_3_-Graphene-CNTs composites, are expected to “extend their application to many new fields as advanced structural materials, protective coatings for micro-mechanical systems and contact-damage-resistant components” [106]. The high hardness and electrical conductivity of Al_2_O_3_-TiC-Ni composites made them excellent candidate materials for manufacturing wear resistant components for many advanced industrial applications [56]. The improved properties and electric conductivity, coupled with enhanced manufacturability of the newly developed electro conductive hybrid nanocomposites, including Al_2_O_3_-SiCw-TiC [54,55] and Al_2_O_3_ reinforced by alumina nanofibers encapsulated by multi-layered graphene [58,59], can greatly improve their quality and reliability, and expand their use in modern high-tech industries. This is of particular importance for applications where products of complex shapes and small sizes are needed.

## 10. Future Directions

The development of alumina hybrid ceramic nanocomposite materials with enhanced comprehensive performance (hardness, fracture toughness, strength, electrical conductivity, thermal conductivity, and thermal stability) is in its early stages and it is expected to continue attracting the interest of the scientific community. Diverse consolidation techniques [151,152,153,154] were used to consolidate alumina hybrid nanocomposites; this review investigated the development of alumina hybrid nanocomposites while using the spark plasma sintering method. These composites have showed their potential as advanced materials that are suitable for new and unique applications. However, “materials discovery today still involves significant trial-and-error. It can require decades of research to identify a suitable material for a technological application, and longer still to optimize that material for commercialization” [155]. Therefore, future research work should be centered on the design and fabrication of alumina hybrid nanocomposites that: (i) are fully dense and free from flaws, (ii) contain the optimum volume fraction of reinforcements, (iii) have uniform distribution of the nanoscale phases; and, (iv) have smooth and strong interface between the reinforcement and matrix. As “the quality of materials determines the success of products and successful products determine the success of a business” [156], the development of high performance alumina hybrid nanocomposite materials that are capable of meeting industrial requirements necessitates addressing the following key points:

(a) Optimization of process parameters of existing synthesis methods such as ball milling [37], sol-gel processing [44], colloidal processing [48], and molecular level mixing [43,133] to not only incorporate the required amount of reinforcements, but to also uniformly disperse the nanoscale phases.
(b) The possibility of introducing innovative nanocomposite powder synthesis techniques needs to be further explored. For instance, the MLM process could be easily used to prepare new hybrid ceramic nanocomposites, including alumina reinforced by carbonaceous materials, such as carbon nanotubes and graphene.
(c) The uniform distribution of the nanoreinforcements in the matrix should be ascertained with the use of advanced and complementary characterization techniques, such as FE-SEM and TEM.
(d) SPS process parameters need to be optimized to prepare fully dense materials that have preserved nanostructures and are free from pores and flaws.
(e) A fundamental understanding of sintering mechanisms is necessary, particularly when the insulating alumina matrix is strengthened by nanoreinforcements that have extremely high electrical and thermal conductivities.
(f) Proper engineering of the interface between the reinforcement and matrix, through careful design of synthesis and processing procedures, is a pre-requisite to have materials with the anticipated properties. This is because the interface influences the load, electron, and phonon transfer among the constituents of the hybrid composite. The quality of the interface needs to be assessed and the interfacial shear stress needs to be measured to have better understating of their influence on the composites’ bulk properties.
(g) Intrinsic and extrinsic strengthening, toughening, and transport mechanisms operating at different length scales and responsible for the change in the properties need comprehensive discussion supported by experimental evidence.
(h) Despite the practical importance of the thermal, electrical, and tribological properties in many applications of alumina hybrid nanocomposites, only limited experimental data are available to date. Therefore, these properties need to be systematically measured and thoroughly understood.
(i) So far, the effect of oxygen stoichiometry on the mechanical and electronic properties has not been investigated. Research work, which would consider this point, is critically needed.
(j) For submicron and nanoscale crystallites, the electrical properties of compounds significantly vary with the same chemical composition; therefore, the effect of the size of real crystallites on the electronic properties of hybrid nanocomposites needs to be investigated.
(k) The fracture toughness of the hybrid nanocomposite materials should be measured while using standard testing methods, such as the single-edged-notched-beam, to obtain reliable data and reduce the discrepancies.

## Figures and Tables

**Table 1:** Properties of the developed Al_2_O_3_-SiC-GNSs composites (Reproduced with permission from [134]. Elsevier, 2019).

Sample	Density (%)	Electrical Conductivity (S/cm)	Grain Size (μm)	Vickers Hardness (GPa)	Fracture Toughness (MPa∙m^0.5^)
Al_2_O_3_ (AO)	>99.0	8.92 ± 3.86 × 10^−13^	5.76 ± 2.38	15.77 ± 0.71	3.30 ± 0.12
Al_2_O_3_-5SiC (AS)	>99.0	1.49 ± 0.29 × 10^−7^	1.24 ± 0.38	19.43 ± 0.65	3.17 ± 0.14
Al_2_O_3_-5SiC-0.5GNSs (ASG1)	>99.0	3.62 ± 1.62 × 10^−7^	1.26 ± 0.43	18.07 ± 0.47	3.51 ± 0.09
Al_2_O_3_-5SiC-1GNSs (ASG2)	>99.0	1.86 ± 0.03	1.23 ± 0.45	17.07 ± 0.26	3.92 ± 0.22

**Table 2:** Predicted effective thermal conductivities of spark plasma sintered alumina hybrid nanocomposites [144].

Sample Composition	Experimental Sample Thermal Conductivity [W/m·K]	Predicted Results with Crystallite Size Effect [W/m·K]	Predicted Results without Crystallite Size Effect [W/m·K]
Al_2_O_3_	34.44	34.06	33.92
Al_2_O_3_-5SiC-1CNT	22.20	27.60	29.99
Al_2_O_3_-5SiC-2CNT	21.33	25.60	29.34
Al_2_O_3_-10SiC-1CNT	17.75	21.38	26.69
Al_2_O_3_-10SiC-2CNT	17.83	19.62	26.30

**Table 3:** Microstructure characteristics and mechanical properties of some spark plasma sintered alumina hybrid nanocomposites.

Materials Composition (%)			SPS Process Parameters				Microstructure Characteristics		Mechanical Properties			Ref.
Al_2_O_3_	First Reinforcement	Second Reinforcement	Heating Rate (deg./min.)	Pressure (MPa)	Temperature (°C)	Time (min)	Relative Density (%)	Alumina Grain Size (μm)	Hardness (MPa)	Fracture Toughness (MPa m ^1/2^)	Flexural Strength (MPa)	-
100	-	-	-	50	1550	-	100	-	˃17	˃3	˃335	[32]
Bal.	1vol.%SiC	5wt.%CNTs	-	50	1550	-	98.2	-	˃16	˃6	˃485	[32]
Bal.	1vol.%SiC	7wt.%CNTs	-	50	1550	-	97.2	-	˃15	˃6	˃480	[32]
Bal.	1vol.%SiC	10wt.%CNTs	-	50	1550	-	95.1	-	˃14	˃5	˃440	[32]
100	-	-	-	50	1550	-	100	-	17	˂4	˃330	[40]
Bal.	1vol.%SiC	5vol.%CNTs	-	50	1550	-	98	-	17	˃6	≈500	[40]
Bal.	2vol.%SiC	5vol.%CNTs	-	50	1550	-	96.46	-	16	≈6	≈450	[40]
Bal.	3vol.%SiC	5vol.%CNTs	-	50	1550	-	96.40	-	16	˂6	≈450	[40]
100	-	-	100	50	1500	10	99.3	-	18.56	3.61	-	[41]
Bal.	5wt.%SiC	1wt.%CNTs	100	50	1500	10	99.36	-	19.77	3.89	-	[41]
Bal.	5wt.%SiC	2wt.%CNTs	100	50	1500	10	98.28	-	19.11	4.2	-	[41]
Bal.	10wt.%SiC	1wt.%CNTs	100	50	1500	10	98.63	-	20.81	4.58	-	[41]
Bal.	10wt.%SiC	2wt.%CNTs	100	50	1500	10	98.02	-	17.50	6.98	-	[41]
100	-	-	100	50	1500	10	99.3		18.56	3.61	-	[43]
Bal.	5wt.%SiC	1wt.%CNTs	100	50	1500	10	91.65		17.81	5.83	-	[43]
100	-	-	100	50	1500	3	100	4.68	18.04	3.53	400	[39]
Bal.	1vol.%SiC	0.38vol.%GPL	100	50	1500	3	99.03	3.67	21.34	4.77	572	[39]
Bal.	3vol.%SiC	0.38vol.%GPL	100	50	1500	3	98.85	2.66	24.65	5.03	520	[39]
Bal.	5vol.%SiC	0.38vol.%GPL	100	50	1500	3	97.35	2.33	21.58	4.94	535	[39]
100	-	-	100	40	1650	10	98	12	13.5	≈4	350	[53]
	0.5wt.%GNT	0.5wt.%GNT	100	40	1650	10	99	-	14.75	5.75	450	[53]
Bal.	0.5wt.%GNT	1wt.%CNT	100	40	1650	10	99	-	15.5	≈4.5	325	[53]
Bal.	1wt.%GNT	1wt.%CNT	100	40	1650	10	98	-	11.20	≈4	340	[53]
Bal.	xvol.%SiC_W_	-	25	30	1780	15	95.53	-	15.85	-	525	[54]
Bal.	xvol.%SiC_W_	22vol.%TiC	25	30	1780	15	99.74	-	21.60	-	648	[54]
42	36%SiC_W_	22%TiC	100	40	1780	5	˃99	-	22.74	6.5	-	[55]
100	-	-	50	100	1375	3	>98	3 ± 1	19.9	3.5	395	[56]
73.1	25vol.% nTiC	1.9vol.% nNi	50	100	1375	3	>98	0.3 ± 0.1	25.6	3.7	537	[56]
70	20vol.%CNF	10vol.%SiC	-	50	1500	3	-	-	-	2.79	144	[57]
